# Knowledge, Attitudes, and Practices (KAP) Regarding Sexually Transmitted Infections, Human Papillomavirus, and HPV-Related Oropharyngeal Cancer Among Italian Adolescents: A Cross-Sectional Study

**DOI:** 10.3390/dj14080486

**Published:** 2026-08-05

**Authors:** Gennaro Musella, Noemi Giorgio, Alejandro I. Lorenzo-Pouso, Lorenzo Lo Muzio, Stefania Cantore, Bojan Poposki, Carlos Gabriel Morales Arenas, Andrea Ballini

**Affiliations:** 1 Department of Clinical and Experimental Medicine, University of Foggia, 71122 Foggia, Italy; gennaro.musella@unifg.it (G.M.); noemi_giorgio.571006@unifg.it (N.G.); lorenzo.lomuzio@unifg.it (L.L.M.); 2 Oral Medicine, Oral Surgery and Implantology Unit (MedOralRes), Faculty of Medicine and Dentistry, Universidad de Santiago de Compostela, 15782 Santiago de Compostela, Spain; alejandro.ismael.lorenzo.pouso@sergas.es; 3 Department of Life Sciences, Health and Health Professions, Link Campus University, 00165 Rome, Italy; a.ballini@unilink.it; 4 Department of Oral and Periodontal Diseases, Faculty of Dentistry-Skopje, Ss. Cyril and Methodius University in Skopje, 1000 Skopje, North Macedonia; bpoposki@stomfak.ukim.edu.mk; 5 Independent Researcher, Santiago de Chile 8320000, Chile; contactocarlosmoralesa@gmail.com

**Keywords:** human papillomavirus, oropharyngeal cancer, oral cancer, sexually transmitted infections, HIV, HPV vaccination, dental public health, oral-health providers, adolescents, cross-sectional study

## Abstract

**Background/Objectives**: Human papillomavirus (HPV) is now responsible for the majority of oropharyngeal squamous cell carcinomas (OPSCCs), an HPV-related cancer of direct relevance to the dental profession; oral-health providers may be well-positioned to perform routine oral examination, recognize suspicious signs, refer appropriately, and educate patients about HPV-related oropharyngeal cancer and HPV vaccination. This study assessed knowledge, attitudes and practices regarding sexually transmitted infections (STIs), HPV, HIV/AIDS and HPV-related oral and oropharyngeal cancer among secondary-school students in Apulia, Italy, with particular attention to the dental setting. **Methods**: A cross-sectional study was conducted by the University of Foggia among 1224 students from three secondary schools at the end of the 2025/2026 school year) using a self-administered 108-item questionnaire. A 63-item composite knowledge score was computed; “don’t know” answers were scored as incorrect and adequate knowledge defined a priori as ≥60% (Bloom’s cut-off). The minimum required sample (737) was derived using Cochran’s formula adjusted for a cluster-sampling design effect. **Results**: Overall knowledge was low (mean 28.5%, SD 18.2); only 5% reached the ≥60% threshold (Bloom’s cut-off). Only 14.8% of students were aware that HPV can cause oropharyngeal cancer, the lowest figure among all oncogenic outcomes assessed and significantly lower in boys (11.2%) than girls (17.3%; *p* = 0.003), despite boys carrying the higher OPSCC risk. Knowledge was independently associated with female sex, age and sexual experience. Despite poor knowledge, 61.2% of students would like to receive the HPV vaccine. **Conclusions**: The dental visit may represent an under-used opportunity to address this oral-cancer-prevention knowledge gap; the integration of oral-health professionals into school-based and clinical HPV-prevention strategies is a plausible public-health implication.

## 1. Introduction

Human papillomavirus (HPV) is the most common sexually transmitted infection worldwide, and the great majority of sexually active individuals come into contact with the virus during their lifetime [1]. While the carcinogenic role of high-risk HPV genotypes in cervical cancer has long been recognized, the epidemiological landscape of HPV-related disease has shifted markedly. Persistent oral infection with high-risk HPV, chiefly HPV16, is now established as the principal cause of a rising subset of oropharyngeal squamous cell carcinomas (OPSCCs) [2,3].

HPV does not, however, act in isolation. Sexually transmitted infections (STIs) as a whole, including chlamydia, gonorrhea, syphilis and HIV/AIDS, represent a major and resurgent public-health problem, with the highest burden concentrated in the 15–24-year age group [4]. In Italy, national surveillance has documented a steady rise in reported STIs in recent years, and adolescents and young adults remain the most affected demographic [5]. Adolescents are biologically and behaviorally vulnerable, and limited knowledge of transmission routes and protective measures is a recognized driver of risk. Because HPV shares both its transmission routes and its target population with other STIs, adolescent knowledge of HPV is best understood against the backdrop of STI knowledge in general, which is the broader domain assessed by the present study.

The incidence of HPV-positive OPSCC has risen to epidemic-like proportions in industrialized countries: the proportion of oropharyngeal cancers staining positive for HPV increased from approximately 16% in the mid-1980s to over 70% in recent United States estimates, with a roughly three-fold increase in HPV-positive OPSCC documented between 2000 and 2017 [3,6]. In several high-income settings, HPV-driven oropharyngeal cancer has overtaken cervical cancer as the most common HPV-associated malignancy. Affected patients tend to be younger, are predominantly male and are frequently never- or former-smokers, distinguishing this entity from the classic tobacco- and alcohol-related head and neck cancers [3,7].

This shift places HPV-related oncology squarely within the remit of the dental profession. The oropharynx, comprising the base of the tongue and the palatine and lingual tonsils, falls within the anatomical field routinely inspected during dental and oral-health visits [7]. Professional dental organizations, including the American Dental Association, have adopted policies urging dentists to support HPV vaccination as a means of preventing HPV-related oropharyngeal cancer, and dedicated action guides for oral-health professionals have been developed [8,9]. Oral-health providers see large numbers of adolescents and young adults for routine, recurring appointments; survey data indicate that most unvaccinated individuals have visited an oral-health provider in the preceding year, underscoring the dental setting as a strategic, under-used venue for HPV-prevention messaging [10].

Nevertheless, the evidence consistently shows that HPV-related discussions are infrequent in dental practice, partly because of gaps in provider knowledge and communication confidence, and that public awareness of the link between HPV, the oral cavity and oropharyngeal cancer is limited [10,11]. Studies of dental students and practitioners have likewise reported incomplete knowledge of HPV-associated OPSCC [12]. Effective deployment of the dental team in oral-cancer prevention requires, as a starting point, an understanding of what young people, the population targeted by HPV vaccination programs, actually know and believe about HPV, oral cancer and the vaccine.

Italy provides a relevant context. HPV vaccination has been actively and freely offered since 2008 and is now recommended for both sexes from the age of 11–12 years; however, national coverage remains below target, with southern regions, including Apulia, historically below the national average [13]. Previous Italian studies among adolescents and young adults have documented limited and partial HPV knowledge, with particular weakness regarding the non-cervical consequences of infection [14,15,16].

Against this background, the present study, conducted by the University of Foggia (Apulia, Italy), had three aims: (i) to assess knowledge of sexually transmitted infections (STIs) in general, including HIV/AIDS, and, specifically, of HPV and HPV-related oral and oropharyngeal cancer among secondary-school students in Apulia; (ii) to describe attitudes towards HPV vaccination and reported sexual and preventive practices; and (iii) to identify the factors associated with knowledge. While the questionnaire covered the broader STI domain, the findings are interpreted primarily from the perspective of the oral-health professional and the dental visit as an opportunity for HPV-related oral-cancer prevention.

## 2. Materials and Methods

### 2.1. Study Design and Population

An observational cross-sectional study was conducted by the University of Foggia among students attending three secondary schools in the Apulia Region (Southern Italy) during the end of the 2025–2026 school year. Within each school all available classes were invited to participate. The inclusion criterion was enrolment in a participating secondary school. An anonymous electronic questionnaire was distributed online to eligible students. Submitted questionnaires that were blank or lacked authorization for data processing were excluded. Participation was voluntary and anonymous. The final analytical sample comprised 1224 valid questionnaires. This study was designed, conducted and reported in accordance with the STROBE (Strengthening the Reporting of Observational Studies in Epidemiology) guidelines for cross-sectional studies; the completed STROBE checklist is provided as a separate Supplementary Material (“STROBE checklist”).

**Figure 1:**
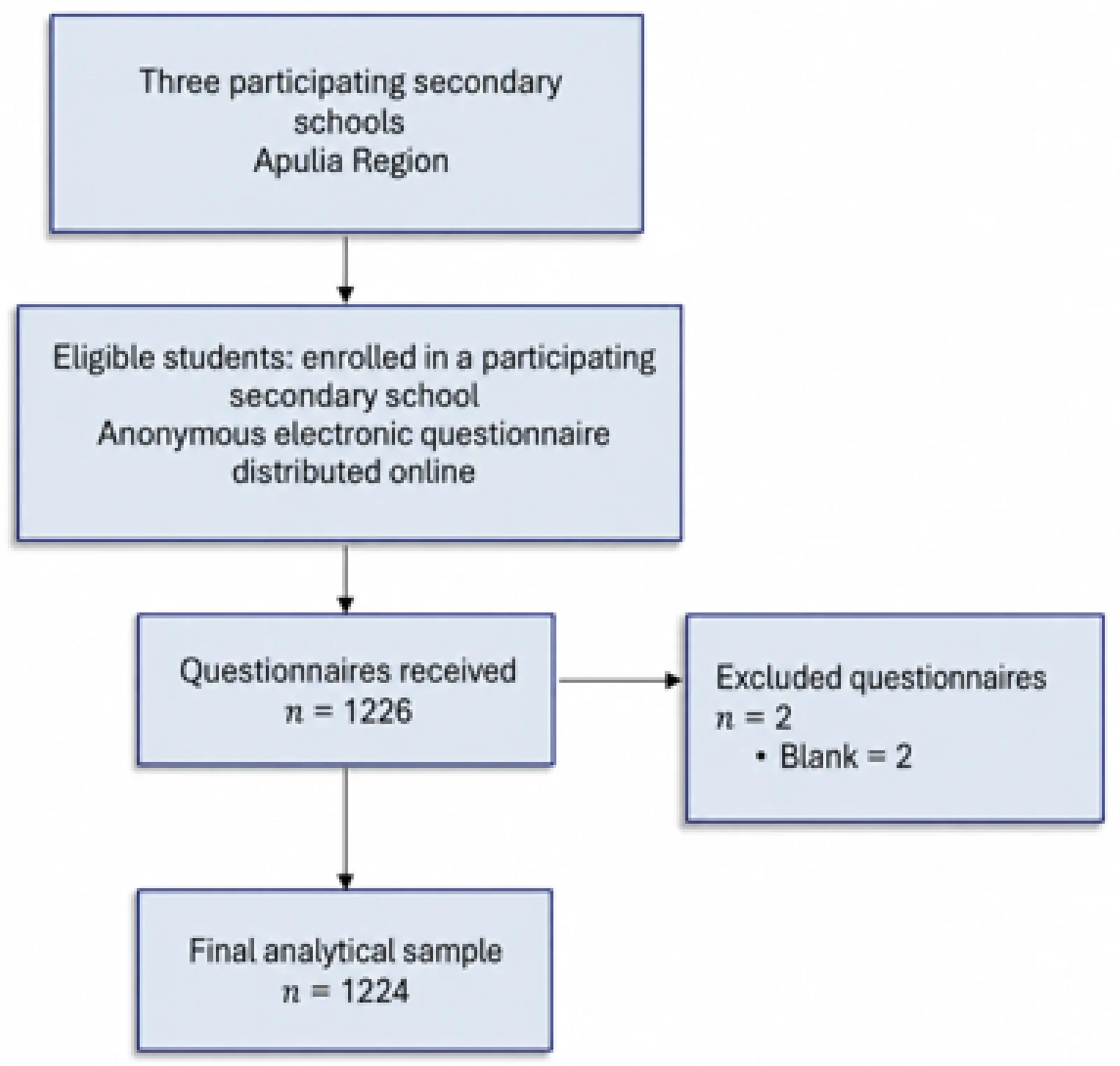
STROBE-style flow diagram of participant recruitment. Eligible students were those enrolled in one of the three participating secondary schools in the Apulia Region during the end of the 2025–2026 school year. An anonymous electronic questionnaire was distributed online; submitted questionnaires that were blank or lacked authorization for data processing were excluded, yielding a final analytical sample of 1224. Note: Because the survey was administered anonymously through the participating schools, the number of eligible students, the number of participating classes, and school-specific response rates were not recorded and are therefore not reported.

The flow of participants from invitation to the final analytical sample is shown in Figure 1 (STROBE-style flow diagram).

### 2.2. Questionnaire

A structured, self-administered questionnaire of 108 items was used and organized into five sections: (i) socio-demographic data (sex, age, school, parental education); (ii) sexual and preventive behaviors; (iii) a 63-item knowledge block on STIs, HPV/HPV vaccination and HIV/AIDS; (iv) attitudes towards HPV vaccination on a three-point ordinal scale; and (v) information sources. The knowledge block included a dedicated item on HPV-related oropharyngeal cancer (“HPV can also cause cancer of the mouth and throat (oropharynx)”), the item of primary interest for the dental perspective of this study, alongside items on cervical and other anogenital HPV-related cancers and on genital warts.

### 2.3. Knowledge Scoring

For each of the 63 knowledge items the correct response was defined a priori from the scientific literature and public-health guidance, and each item was recoded as 1 (correct) or 0 (incorrect). Consistent with established practice in knowledge–attitudes–practices (KAP) and cancer-knowledge research, “don’t know” responses were scored as incorrect, as they denote absence of knowledge rather than its presence [17,18]. An overall score and three subscale scores (general STIs, 40 items; HPV/vaccine, 14 items; HIV/AIDS, 9 items) were computed as the percentage of correct responses. Adequate knowledge was defined a priori as an overall score ≥ 60%, corresponding to the lower bound of Bloom’s widely used cut-off points (poor < 60%, moderate 60–79%, good ≥ 80%) and the threshold most frequently adopted to dichotomize knowledge in KAP studies [19,20].

The continuous overall score and the three domain scores were treated as the primary knowledge outcomes. The ≥60% dichotomization (Bloom’s cut-off) was retained as a secondary, descriptive endpoint for comparability with previous KAP studies and is not intended as a validated clinical threshold; students scoring below it are described as scoring “below the 60% threshold” rather than as having objectively inadequate knowledge. The full item-level answer key, scoring rules and a reproducible analysis script are provided in Supplementary Material S1.

### 2.4. Questionnaire Development, Cross-Cultural Adaptation and Content Validation of the Instrument

The knowledge items were assembled through a two-stage process. In the first stage, a pool of candidate items was compiled from previously published instruments assessing knowledge of HPV, HPV-related oropharyngeal cancer, HIV/AIDS and other sexually transmitted infections among adolescents and young people [21,22,23,24,25]. From this pool, the authors removed items that were redundant across instruments, items whose original response format (e.g., self-rated familiarity, multiple-choice or open-ended questions) was incompatible with the dichotomous true/false format adopted here, and items that, although relevant, were set aside to limit questionnaire length and respondent burden in a classroom setting. Items addressing the HPV–oropharyngeal-cancer association, central to the dental focus of the study, were adapted from the corresponding item of the French school-staff instrument [23] and extended to specify the oropharynx. The source mapping, together with the full candidate pool and the reasons for inclusion or exclusion, is provided in Supplementary Material S2.

Because the source items had been developed in English, a formal cross-cultural adaptation into Italian was carried out following the internationally recommended guideline of Sousa and Rojjanasrirat for the translation and validation of instruments in cross-cultural health-care research [26]. The procedure comprised six stages: (i) two independent forward translations into Italian by bilingual translators, one with and one without a health-care background; (ii) synthesis of the two forward translations into a single reconciled version, with disagreements resolved by discussion; (iii) two independent back-translations into English by further bilingual translators blinded to the original items; (iv) comparison of the back-translated versions with the source instrument to detect and correct semantic, idiomatic, experiential and conceptual discrepancies; (v) review of the pre-final Italian version by the expert committee; and (vi) pilot testing on the target population. This forward–backward methodology mirrors that adopted by comparable Italian HPV studies conducted in the dental field [27], ensuring conceptual, semantic, idiomatic and content equivalence between the Italian and English versions.

In the second stage, content validity was assessed by an expert panel of five members: three oral-medicine physicians (two with specific research expertise in knowledge–attitudes–practices studies on HPV), one dentist with clinical experience in oral medicine, and one educationalist (pedagogist) with expertise in adolescent health education. In addition, an infectious-disease specialist was consulted to review the clinical accuracy of the STI and HIV/AIDS items; this consultation was qualitative and did not contribute to the quantitative ratings. Each of the five panel members independently rated every item for relevance on a four-point ordinal scale (1 = not relevant to 4 = highly relevant). The item-level content validity index (I-CVI) was computed as the proportion of experts rating an item 3 or 4, the scale-level index (S-CVI/Ave) as the mean of the I-CVI values, and the modified kappa (κ*) as the chance-corrected agreement [28]. Items were retained when I-CVI ≥ 0.78, the threshold recommended for panels of three to five experts [29]. Retained items showed I-CVI values ranging from 0.80 to 1.00 (S-CVI/Ave = 0.99) and modified kappa from 0.76 to 1.00, indicating good-to-excellent agreement beyond chance; four candidate items fell below the threshold and were excluded. The item-level I-CVI and kappa values are reported in full in Supplementary Material S2.

The comprehensibility, face validity and completion time of the pre-final Italian version were verified through a pilot test on 30 students who were not included in the final analysis; the mean completion time was 15–20 min and no item was reported as unclear. Cronbach’s alpha values ≥ 0.70 were considered indicative of acceptable internal consistency. While the overall questionnaire demonstrated high internal consistency (α = 0.93), the HIV/AIDS subscale showed only moderate internal consistency (α = 0.65), which should be interpreted with caution. The high alpha value for the overall questionnaire may also partly reflect the relatively large number of items included. Moreover, internal consistency represents only one aspect of questionnaire evaluation and does not establish construct validity, criterion validity, test–retest reliability, or responsiveness. The complete questionnaire, together with the distribution of responses for the knowledge items, is provided in Supplementary Material S3.

### 2.5. Statistical Analysis

Categorical variables are reported as frequencies and percentages and quantitative variables as mean and standard deviation (SD) or median. The analysis followed a pre-specified plan. The primary outcomes were the continuous overall knowledge score and the three domain scores; the secondary outcome was the dichotomized ≥60% adequacy endpoint; item-level and parental-education analyses were exploratory.

The distribution of the continuous score was inspected for normality and homogeneity of variance was assessed with Levene’s test. Two-group score comparisons used Student’s *t*-test, with Welch’s correction when variances were unequal; comparisons across more than two groups used one-way ANOVA with Tukey’s post hoc test, with the Kruskal–Wallis test reported as a non-parametric confirmation. Associations between categorical variables used Pearson’s chi-square test with Cramér’s V, and correlations used Pearson’s or Spearman’s coefficient as appropriate. Effect sizes were quantified with Cohen’s d and eta-squared [30].

A multivariable linear regression was fitted for the continuous overall score (primary outcome), and multivariable logistic regression for the ≥60% adequacy endpoint and for correct awareness of HPV-related oropharyngeal cancer, reporting odds ratios (ORs) with 95% confidence intervals (CIs). Item-level comparisons were treated as exploratory and adjusted for multiplicity using the Benjamini–Hochberg false-discovery-rate procedure (Supplementary Material S4). Because the ≥60% adequacy outcome was rare, multicollinearity was assessed using variance inflation factors and the logistic model was re-estimated with Firth’s penalized-likelihood method to guard against sparse-data bias, complemented by a sensitivity analysis using a less extreme ≥50% threshold. A two-sided *p* < 0.05 was considered significant. Analyses were performed in Python 3.12 (pandas, SciPy, statsmodels, pingouin).

### 2.6. Influence of Parental Education

A pre-specified secondary objective was to assess whether parental educational level influences adolescents’ knowledge and attitudes, given evidence that the family socio-cultural context may shape adolescent health literacy and that some Italian studies have reported better HPV knowledge among adolescents with more educated parents [31,32]. Parental education was recorded for the mother and the father separately and categorized into four ordinal levels (primary-school certificate, lower-secondary certificate, upper-secondary diploma, university degree); the higher of the two parents was also used as a composite indicator. The association of parental education with the overall knowledge score and with a pro-vaccination attitude score (mean of three vaccine-attitude items, range 1–3) was tested using one-way ANOVA, the Kruskal–Wallis test and Spearman’s correlation, and was confirmed in a multivariable logistic model adjusted for sex, age and school-based sex education.

### 2.7. Size Calculation

The required sample size was estimated for the primary descriptive objective, namely estimating the prevalence of adequate knowledge, using Cochran’s formula for a single proportion, n_0_ = Z^2^ × p × (1 − p)/d^2^ [33,34], with a 95% confidence level (Z = 1.96), an absolute precision of ± 5% (d = 0.05) and, in the absence of a prior estimate, the most conservative prevalence *p* = 0.50 (maximizing variance), n_0_ ≈ 385. To account for class-based cluster sampling, this was inflated by a design effect DEFF = 1 + (m − 1) × ICC, assuming a mean class size m = 22 and a conservative intra-class correlation ICC = 0.03, DEFF = 1.63, giving 627. A further 15% inflation for non-response yielded a minimum required sample of approximately 737. The achieved sample of 1224 comfortably exceeds this requirement, corresponding to an effective absolute precision of approximately ±3.6% for a 50% prevalence estimate. This calculation establishes the precision of the prevalence estimate for the achieved sample; it does not confer regional representativeness, since the three schools were selected by convenience rather than by probability sampling (see Limitations). The calculation is summarized in Table 1.

### 2.8. Ethics

The study was conducted in accordance with the principles of the Declaration of Helsinki and was approved by the Ethics Committee of the University of Foggia (Prot. n. 0037659-III/13; date of approval: June 2026). The authorization was obtained from the participating schools. Electronic informed consent was obtained from the parents or legal guardians of participants younger than 18 years of age, and electronic assent was obtained from the adolescents before questionnaire administration, in accordance with the study protocol approved by the Ethics Committee.

## 3. Results

### 3.1. Sample Characteristics and Behaviors

A total of 1224 valid questionnaires were analyzed. Girls accounted for 56.1% (*n* = 687) and boys for 43.1% (n = 528); mean age was 16.15 years (SD 1.52; range 13–19). Most students attended a general secondary school (52.5%) or a technical secondary school (42.9%). Regarding behavior, 37.1% reported having had sexual intercourse and 29.7% being currently sexually active; only 22.0% reported having received any sex education at school. Socio-demographic and behavioral characteristics are summarized in Table 2.

### 3.2. Overall Level of Knowledge

**Figure 2:**
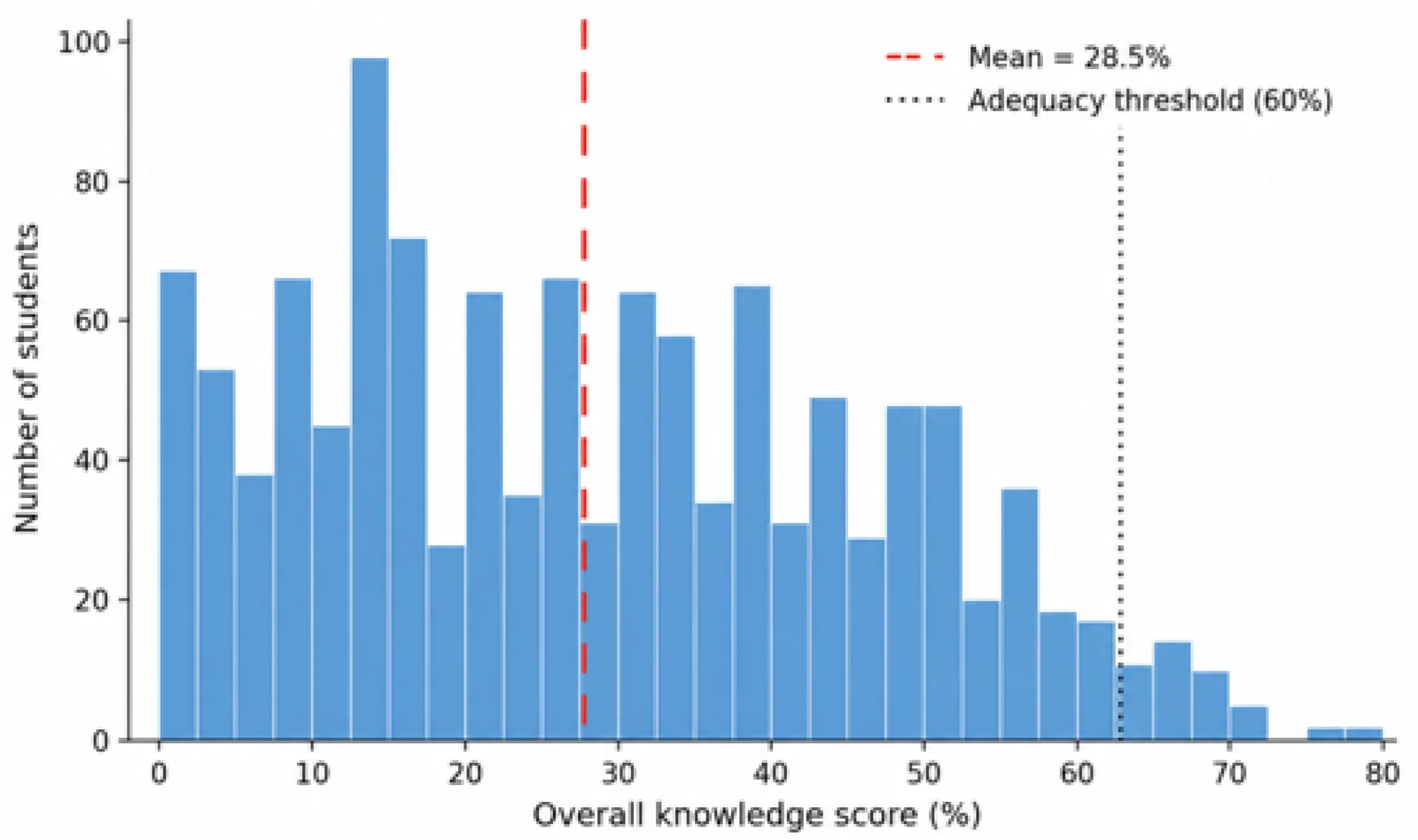
Distribution of the overall knowledge score (N = 1224). The dashed line indicates the sample mean; the dotted line indicates the 60% adequacy threshold.

The overall knowledge score was low: students answered correctly a mean of 28.5% of items (SD 18.2; median 27%). The distribution was right-skewed, and only 5% of students reached the pre-defined adequacy threshold of ≥60% (Figure 2). In sensitivity analyses using alternative thresholds, 11.1% and 0.3% of students reached the ≥50% and ≥70% cut-offs, respectively; the ranking of the three knowledge domains was unchanged across all thresholds (Figure 2). By subscale, knowledge of HIV/AIDS was highest (40.5%), followed by general STIs (26.4%) and HPV/vaccine (26.7%); none reached the 60% threshold. Internal consistency was high (Cronbach’s α = 0.93; see “Limitations” paragraph).

### 3.3. Knowledge of HPV-Related Oral and Oropharyngeal Cancer

**Figure 3:**
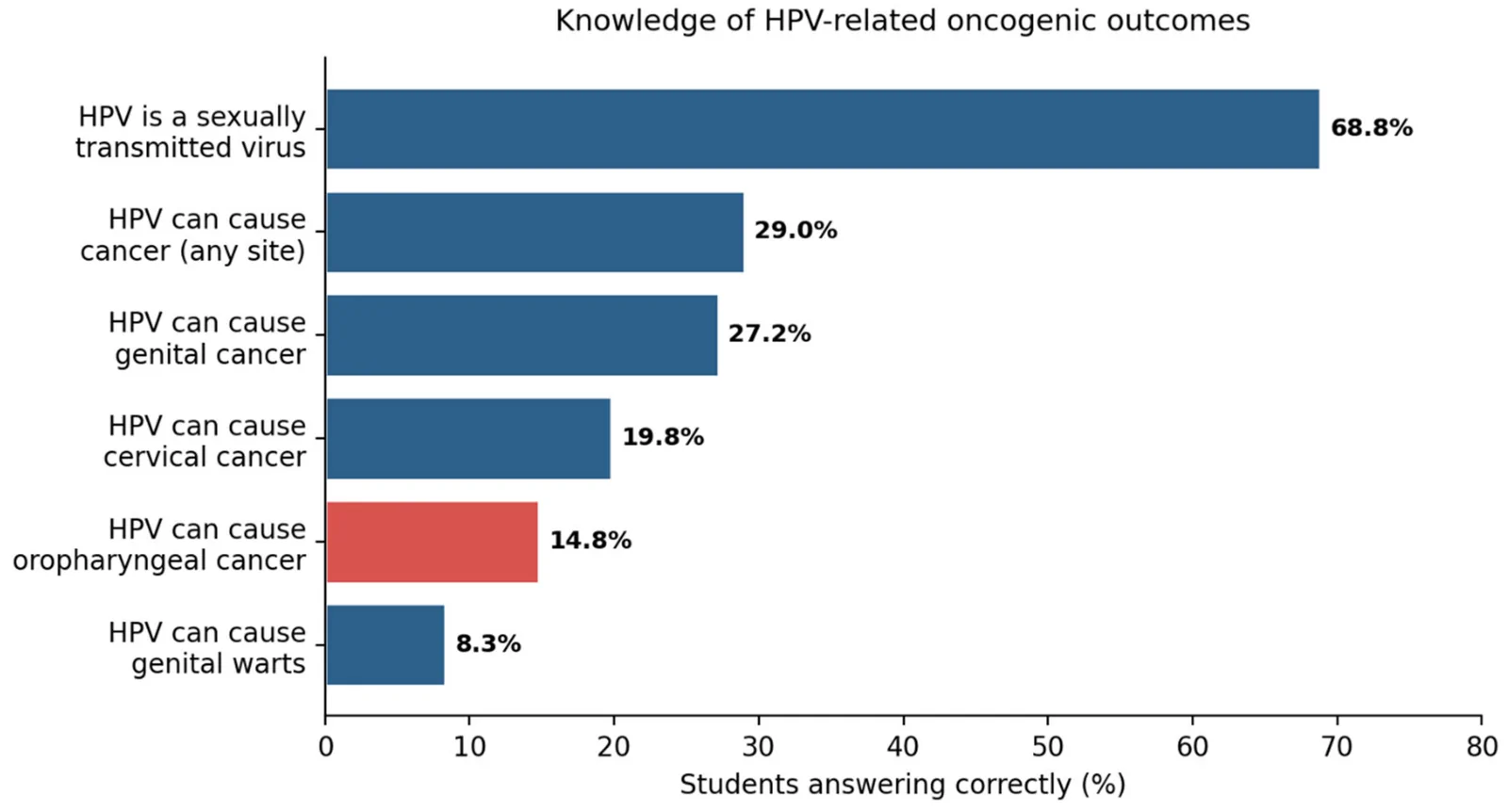
Knowledge of HPV-related oncogenic outcomes (% of students answering correctly). Awareness of HPV-related oropharyngeal cancer (highlighted) was the lowest among all malignancy-related items assessed.

The findings of greatest relevance to the dental setting concern knowledge of the oncogenic consequences of HPV infection (Figure 3). Although recognition of HPV as a sexually transmitted virus was comparatively common (68.8%), awareness of its cancer-causing potential was strikingly limited: only 29.0% of students knew that HPV can cause cancer at all. Knowledge of specific HPV-related cancers was uniformly poor, and awareness of HPV-related oropharyngeal cancer was the lowest of all: only 14.8% of students correctly answered that HPV can cause cancer of the mouth and throat. This was lower than the proportion aware of HPV-related cervical cancer (19.8%) and of genital cancer in general (27.2%).

For the oropharyngeal-cancer item, the great majority of students (973/1224, 79.5%) answered “don’t know”, indicating widespread unawareness rather than a definite incorrect belief. Awareness of HPV-related oropharyngeal cancer was significantly lower in boys than girls (11.2% vs. 17.3%; χ^2^ = 11.43; *p* = 0.003; Benjamini–Hochberg FDR-adjusted *p* = 0.012). This sex gap is of particular public-health concern, because HPV-positive oropharyngeal cancer disproportionately affects men.

In a multivariable logistic regression, correct awareness of HPV-related oropharyngeal cancer was independently associated with older age (OR 1.56 per year, 95% CI 1.37–1.78; *p* < 0.001) and female sex (OR 1.48, 95% CI 1.05–2.10; *p* = 0.025), whereas school type, receipt of sex education and sexual experience were not (Table 3).

### 3.4. Misconceptions

Item-level analysis identified several persistent misconceptions and areas of weak knowledge. The most striking was that HPV affects only women, correctly rejected by only 7.3% of students, a misconception directly relevant to the under-recognition of oropharyngeal-cancer risk in males. Knowledge of the modes and limits of STI protection was also incomplete: only 45.0% of students correctly recognized that condoms do not offer 100% protection, and just 16.9% knew that oral contraceptives do not reduce STI risk, indicating a widespread overestimation of protective measures. By contrast, more than half correctly understood that infections such as HIV are not transmitted through food sharing (56.0%). The complete item-by-item distribution of correct, incorrect and “don’t know” responses for all 63 knowledge items, as well as the full questionnaire, is reported in Supplementary Material S3.

### 3.5. Factors Associated with Overall Knowledge

In bivariate analyses, overall knowledge was higher in girls than boys (30.5% vs. 25.9%; t = 4.47; *p* < 0.001; Cohen’s d = 0.25), increased with age (Pearson’s r = 0.32; *p* < 0.001) and was higher among students who had received sex education (31.2% vs. 27.8%; *p* = 0.009) and those reporting sexual experience (33.6% vs. 25.6%; *p* < 0.001). No difference emerged across school types (ANOVA F = 1.08; *p* = 0.354) and no association with parental education (Spearman’s ρ = −0.020; *p* = 0.497).

In the multivariable linear regression on the continuous overall score (the primary outcome; n = 1213; R^2^ = 0.13), higher knowledge was independently associated with older age (β = +3.42 per year, 95% CI +2.69 to +4.14; *p* < 0.001), sexual experience (β = +4.47, 95% CI +2.27 to +6.67; *p* < 0.001), female sex (β = +3.84, 95% CI +1.88 to +5.80; *p* < 0.001) and receipt of school sex education (β = +3.95, 95% CI +1.62 to +6.28; *p* = 0.001), whereas attending a general secondary school (*p* = 0.056) and parental education (*p* = 0.152) were not. In the secondary multivariable logistic model for an overall score ≥60% (Table 4; 61 events), older age remained independently associated with adequate knowledge (OR 1.81 per year; *p* < 0.001), whereas female sex and sexual experience, although associated with the continuous score, were attenuated and no longer statistically significant after adjustment (OR 1.61, *p* = 0.110; and OR 1.45, *p* = 0.221, respectively); high-school attendance and receipt of school sex education were also non-significant. Multicollinearity was negligible (all variance inflation factors < 1.4), and a Firth penalized-likelihood re-estimation yielded consistent results, confirming that the attenuation was not due to the rarity of the outcome. The weaker associations in this model are consistent with the reduced power of the rare dichotomized outcome relative to the continuous score.

### 3.6. Influence of Parental Education on Knowledge and Attitudes

Parental educational level showed no consistent association with adolescents’ knowledge or attitudes (Table 5). For the overall knowledge score, one-way ANOVA using the higher parental education level was nominally significant (F = 4.79; *p* = 0.003); however, this result was entirely driven by the small primary-school subgroup (n = 10), whose mean score (48.3%) was an outlier. When this subgroup was excluded, the association disappeared (F = 1.29; *p* = 0.276), and Tukey’s post hoc test identified significant differences only for comparisons involving the primary-school subgroup. Spearman’s correlation, which is insensitive to such small-group artifacts, indicated no monotonic relationship between parental education and knowledge (ρ = −0.020; *p* = 0.497). In the multivariable logistic model adjusted for sex, age and school sex education, parental education was not independently associated with adequate knowledge (OR = 0.97, 95% CI 0.67–1.42; *p* = 0.893).

Parental education was likewise unrelated to the pro-vaccination attitude score: no significant difference emerged across categories of maternal education (ANOVA F = 0.6; *p* = 0.613), paternal education (F = 1.43; *p* = 0.234) or the composite indicator (F = 1.22; *p* = 0.303; Spearman’s ρ = 0.014; *p* = 0.621). Overall, in this sample the family’s educational background did not measurably shape either the knowledge or the attitudes of adolescents regarding HPV.

### 3.7. Attitudes Towards HPV Vaccination and Information Sources

Despite the poor knowledge profile, attitudes towards HPV vaccination were favorable: 61.2% of students would like to receive the vaccine and 59.5% agreed that its protective effect is an incentive to be vaccinated. Notably, willingness to be vaccinated was almost identical (59.5%) among the subgroup of students who did not know that HPV can cause oropharyngeal cancer, indicating a receptive audience that simply lacks the oral-cancer-prevention rationale. About 37.8% reported having already been vaccinated (self-reported, not verified coverage), with higher reported coverage in girls (43.8%) than boys (30.5%). Willingness to be vaccinated differed by vaccination status: 82.9% among already-vaccinated students and 48.0% among the unvaccinated. A large majority expressed an explicit information need (74.3% about HPV, 70.6% about the vaccine).

Regarding information sources, the internet was the most frequently cited channel (718 mentions), followed by school (522) and friends (354). Health professionals were marginal: the family doctor was cited 147 times and information leaflets 116 times; the dental visit did not feature as a recognized source of HPV information.

## 4. Discussion

This cross-sectional study of 1224 secondary-school students in Apulia identified a pronounced and clinically relevant gap: awareness of HPV-related oropharyngeal cancer was the weakest of all the oncogenic outcomes assessed, with only 14.8% of students aware of it. This finding is of direct relevance to the dental profession, increasingly recognized as a potential front-line actor in HPV-related oral-cancer prevention [8,9,10], although the present design does not allow the effectiveness of dental-based prevention to be assessed.

### 4.1. A Blind Spot Precisely Where the Dental Team Operates

The epidemiological transition of HPV-positive oropharyngeal cancer, now responsible for the majority of oropharyngeal malignancies in industrialized countries and still rising [3,6], has not been matched by public awareness. In our sample, students were markedly less likely to be aware about HPV-related oropharyngeal cancer (14.8%) than about HPV-related cervical cancer (19.8%), even though both figures are low. The oropharynx is part of the anatomical region routinely examined during dental check-ups, and 40–43% of patients with oral or oropharyngeal cancer symptoms first seek care from a dentist [12,35]; patients also increasingly report dentists as an important source of information on HPV-associated OPSCC [13]. The near-absence of the dental visit among the information sources cited by students therefore represents a missed prevention opportunity rather than an irrelevance.

The observed sex difference is especially salient. Boys knew significantly less than girls about HPV-related oropharyngeal cancer (11.2% vs. 17.3%; *p* = 0.003), yet HPV-positive oropharyngeal cancer disproportionately affects men [3,7]. The historically female-oriented framing of HPV, as a “cervical” and therefore “women’s” issue, is reflected in the widespread misconception in our sample that HPV affects only women (only 7.3% correct). Oral-health messaging directed at male adolescents and their parents could help correct this asymmetry, consistent with the universal (both-sex) HPV vaccination recommendation in Italy [13].

### 4.2. Poor Knowledge, Favorable Attitudes: An Actionable Combination

As in previous Italian studies reporting limited HPV knowledge among young people [14,15,16], overall knowledge was low (mean 28.5%; only 5.0% reaching the ≥60% threshold). Yet attitudes towards vaccination were favorable overall (61.2% would like to be vaccinated), although willingness was more modest among unvaccinated students (48.0%), the group relevant to future uptake, indicating that a substantial minority remains hesitant. Notably, willingness was essentially unchanged among those unaware of the oropharyngeal-cancer link. This dissociation between poor factual knowledge and positive attitudes, accompanied by an explicit demand for information, represents an opportunity rather than an obstacle. For a large part of this audience, oral-health professionals are not required to overcome entrenched ideological resistance but rather to supply a missing, specifically oral, prevention rationale; among the more hesitant minority, the same recurring contact provides repeated opportunities to build confidence over time.

A strong provider recommendation is among the most influential determinants of vaccine uptake [10,36], and the recurring, longitudinal nature of dental care makes the dental team well placed to deliver it.

The knowledge gap was not confined to HPV. The general-STI subscale, the largest component of the instrument with 40 items, recorded the lowest mean score of the three domains (24.9%), and the HIV/AIDS subscale, although the highest (34.4%) still fell well below the adequacy threshold. Persistent gaps in the understanding of protective measures were also apparent: a substantial minority of students overestimated the protection offered by condoms or believed that oral contraceptives reduce STI risk, misconceptions that may generate false reassurance and discourage genuinely protective behavior. This broad fragility of STI knowledge is consistent with the resurgent STI burden documented among Italian adolescents [5] and reinforces the case for comprehensive sexual-health education. For the dental team, it is a reminder that the conversation prompted by the oral-cancer–HPV link can serve as an entry point to a wider, much-needed dialog on sexual health. This is especially pertinent for oral-health professionals, because many STIs, including non-viral pathogens acquired through oral sex, produce oral manifestations that the dental team is well placed to recognize [37,38], reinforcing the value of an evidence-based approach to dental practice [39].

### 4.3. Parental Education Did Not Shape Adolescents’ Knowledge or Attitudes

A pre-specified secondary analysis examined whether the family’s educational background influences adolescents’ HPV knowledge and attitudes. We found no genuine association with either outcome. The only nominally significant result, an ANOVA on the highest parental education level, was an artifact of a 10-student primary-school subgroup and did not survive exclusion of that subgroup, non-parametric testing or multivariable adjustment. This finding contrasts with some Italian and international evidence: a recent Italian study reported that having at least one university-educated parent was associated with higher HPV knowledge and lower vaccine-related anxiety in adolescents [31], and socioeconomic gradients in HPV awareness have been described elsewhere [32]. Several explanations are plausible. First, our sample was older (mean 16 years) than the 11–13-year-olds in whom parental influence on health knowledge is typically strongest, and adolescents increasingly draw HPV information from peers and the internet rather than from parents, which is consistent with our finding that the internet was the dominant information source. Second, the uniformly low knowledge across the whole sample left little variance for parental education to explain. Third, parental education is an imperfect proxy for the family’s specific HPV health literacy. Whatever the explanation, the practical implication reinforces the central message of this study: because the family does not appear to compensate for the knowledge gap, structured external sources of information, namely the school and the dental team, become correspondingly more important.

### 4.4. Implications for the Dental Setting

The findings point to several possible avenues for oral-health professionals and dental public health, which should be read as hypotheses to be tested rather than as evidence-based recommendations. It should be stated explicitly that the present study assessed knowledge, attitudes and practices only: it did not evaluate the effectiveness, feasibility, reach or acceptability of dental-based HPV counseling, and no inference about the impact of such counseling can be drawn from these data. With this caveat in mind, one plausible avenue is the integration of HPV-OPSCC counseling into routine dental visits, linking the extra- and intraoral examination to a brief, standardized explanation of the connection between HPV and oropharyngeal cancer. This could be extended to explicit HPV-vaccination information for adolescents and their parents, framing the vaccine as a form of oral-cancer prevention and emphasizing that the recommendation applies to both sexes. Such messaging might usefully address male adolescents in particular, who in our sample knew significantly less about HPV-related oropharyngeal cancer despite carrying the higher risk. Beyond the dental chair, the involvement of the dental team in school-based health-education programs could be explored, since the school reaches adolescents comprehensively whereas only 22% of students in this study reported having received any sex education. Any such clinical and public-health role would also depend on strengthened undergraduate and continuing dental education on HPV-OPSCC and patient-communication skills, given that gaps in provider knowledge and communication confidence are recognized barriers to HPV discussion in dental practice. While these directions are consistent with the policies of major dental organizations and with dedicated action guides for oral-health professionals [8,9], their effectiveness, feasibility and acceptability in routine practice remain to be established and should be the object of dedicated implementation and health-services research before being adopted as prevention policy. These educational needs do not appear to be specific to the Italian setting. Two recent cross-sectional analyses of the same large cohort of medical-science students at the University of Novi Sad, Serbia (n = 1760, surveyed in November 2023), offer a useful regional comparison. Denda et al. reported a median knowledge score of 6 out of 10, persistent misconceptions about transmission and an explicit wish for further information expressed by more than 60% of respondents [40]; in the companion report, Maletin et al. found that only about one quarter of these students had attended any formal education on HPV in the preceding year, that those who had achieved substantially higher knowledge scores, and that previous education was also associated with a greater willingness to recommend vaccination to future patients [41]. The configuration we observe in Apulian adolescents, limited factual knowledge coexisting with favorable attitudes and an explicit demand for information, thus recurs among older, health-oriented students in a neighboring European setting, and reaches those who will themselves become health professionals and prospective counselors. This comparison should nevertheless be interpreted with caution: the Serbian studies surveyed university students in the medical sciences using a different instrument and scoring system, so absolute percentages are not directly comparable with ours, and the two reports draw on a single survey population. Neither study focused specifically on the oral and oropharyngeal dimension of HPV-related disease, which remains under-explored in the region and which the present study addresses. The convergence of the qualitative pattern across these different populations is nonetheless notable. This strengthens the case for reinforcing HPV content within health-professional curricula, including dental curricula, as a prerequisite for any expanded counseling role.

### 4.5. Limitations

Several limitations should be acknowledged. First, the cross-sectional design precludes causal inference. Second, the three schools were purposively selected and not probabilistically sampled at the regional level, and the sample was unbalanced by school type and sex, limiting generalizability. Therefore, the reported prevalence estimates should be interpreted as applying only to the participating schools and should not be extrapolated to all adolescents in the Apulia Region. Third, behavioral and vaccination data were self-reported and subject to social-desirability and recall bias. Fourth, scoring “don’t know” responses as incorrect, although standard and conservative, mechanically lowers scores; a sensitivity analysis treating them as missing would yield higher estimates. Fifth, parental education was self-reported by adolescents and the primary-school subgroup was very small, limiting the power to detect a true gradient at the lowest educational level. Sixth, the measure of school-based sex education was based on a single yes/no item that did not define the provider, format, or time frame of such education, which may have led to inconsistent interpretation. Although students were recruited through a class-based sampling strategy, the limited number of participating schools and the lack of class-level identifiers in the anonymized dataset precluded reliable cluster-adjusted analyses. The inferential results should therefore be interpreted cautiously, as within-class correlation may have led to underestimated standard errors. Furthermore, although internal consistency was assessed using Cronbach’s alpha, which is equivalent to KR-20 for dichotomously scored knowledge items [42], future psychometric evaluations should also consider reporting McDonald’s omega to provide a more comprehensive assessment of reliability [43]. Although content validity was formally assessed by a five-member expert panel using item-level and scale-level content validity indices, the panel did not include formal expertise in psychometrics or epidemiology; future validation studies should incorporate these competencies, together with cognitive interviewing and additional forms of validity testing.

Finally, awareness of the association between HPV and oropharyngeal cancer was assessed using a single item; future dental-focused instruments should explore this domain in greater depth.

## 5. Conclusions

Among Apulian secondary-school students, awareness of HPV-related oropharyngeal cancer was strikingly low, the weakest of all oncogenic outcomes assessed, and lowest in boys, the group at greater risk. This knowledge gap coincides precisely with the anatomical and preventive remit of the dental team. Combined with the favorable vaccination attitudes and explicit demand for information observed, the findings identify the oral-health setting as a plausible, but as yet untested, venue for HPV-related prevention counseling. It should be emphasized that this study documents a knowledge gap and does not test whether counseling delivered by the dental team is effective, feasible, far-reaching or acceptable; the integration of oral-health professionals into HPV-related oral-cancer prevention, both in the dental chair and within school-based health-education programs, therefore remains a public-health implication that requires dedicated implementation research. Should such research prove supportive, equipping the dental team to deliver clear, both-sex, oral-cancer-framed HPV messaging could represent a high-reach and plausibly low-cost strategy to address an under-recognized and rising cancer burden.

## Figures and Tables

**Table 1:** Sample-size calculation. Required sample size for the prevalence estimate. The achieved sample (1224) exceeds the minimum requirement (737), giving an effective precision of approximately ±3.6%.

Parameter	Value
Confidence level (Z)	95% (1.96)
Expected prevalence (*p*)	0.50
Absolute precision (d)	±5%
Base sample (Cochran, n_0_)	385
Design effect (DEFF)	1.63
Adjusted for clustering	627
Non-response inflation	+15%
Minimum required sample	737
Achieved sample	1224

**Table 2:** Characteristics of the study sample. Socio-demographic and behavioral profile of the participants (*N* = 1224). Condom use is computed among students who reported ever having had sexual intercourse (*n* = 454).

Variable	N.	%
**Sex**		
Girls	687	56.1
Boys	528	43.1
Not specified	9	0.7
**School type**		
General secondary school (Liceo)	643	52.5
Technical secondary school	525	42.9
Vocational secondary school	44	3.6
Vocational training center	12	1.0
**Behaviors**		
Has had sexual intercourse	454	37.1
Currently sexually active	364	29.7
Has heard of STIs	1152	94.1
Received sex education at school	269	22.0
Habitually uses condoms	327	72.0

**Table 3:** Variables independently associated with oropharyngeal-cancer awareness. Multivariable logistic regression.

Variable	OR (95% CI)	*p*
Female sex	1.48 (1.05–2.10)	0.025
Age (per year)	1.56 (1.37–1.78)	<0.001
General secondary school	0.82 (0.57–1.18)	0.284
School sex education	1.17 (0.79–1.73)	0.426
Sexual experience	0.85 (0.59–1.23)	0.385

**Table 4:** Variables independently associated with an overall knowledge score ≥60%. Multivariable logistic regression (n = 1213; 61 events). Estimates were confirmed by Firth penalized-likelihood regression. McFadden’s pseudo-R^2^ = 0.143.

Variable	OR (95% CI)	*p*
Age (per year)	1.81 (1.44–2.27)	<0.001
Female sex	1.61 (0.90–2.89)	0.11
Sexual experience	1.45 (0.80–2.62)	0.221
General secondary school	0.63 (0.31–1.25)	0.183
School sex education	1.61 (0.89–2.94)	0.117
Parental education	1.07 (0.72–1.58)	0.746

**Table 5:** Knowledge and pro-vaccination attitude scores by parental educational level.

Parental Education	Knowledge (%)	Attitude (1–3)	N.	p (Knowledge) ^1^	p (Attitude) ^1^
**Maternal education**				0.118	0.613
Primary	37.2	2.51	24		
Lower-secondary	28.5	2.54	293		
Upper-secondary	28.9	2.56	525		
University	28.0	2.51	364		
**Paternal education**				0.040	0.234
Primary	38.8	2.68	26		
Lower-secondary	28.5	2.52	351		
Upper-secondary	28.3	2.56	534		
University	28.6	2.51	292		
**Highest parental level**				0.003	0.303
Primary	48.3	2.77	10		
Lower-secondary	27.3	2.50	185		
Upper-secondary	29.3	2.55	563		
University	27.8	2.53	456		

^1^ *p*-values are for the overall comparison across educational levels within each block (one-way ANOVA), for the knowledge score and the pro-vaccination attitude score, respectively. The nominal significance for the highest parental level is an artifact of the small primary-school subgroup (n = 10); it disappears on exclusion of that subgroup (*p* = 0.276) and is not confirmed by Spearman’s correlation or by the adjusted logistic model.

## Data Availability

The data presented in this study are available on request from the corresponding author. The data are not publicly available due to privacy restrictions.

## Supplementary Materials

The following supporting information can be downloaded at: https://www.mdpi.com/article/10.3390/dj14080486/s1. Supplementary Material S1: Codebook and reproducible analysis code; Supplementary Material S2: Questionnaire development and content validation; Supplementary Material S3: Full questionnaire and distribution of responses; Supplementary Material S4: Item-level comparison of correct-response rates by sex, with FDR-adjusted *p*-values; STROBE Statement—checklist of items that should be included in reports of observational studies.
