# Supplementary material for: Knowledge, Attitudes, and Practices (KAP) Regarding Sexually Transmitted Infections, Human Papillomavirus, and HPV-Related Oropharyngeal Cancer Among Italian Adolescents: A Cross-Sectional Study
Source: Dent J (Basel). 2026 Aug 5;14(8):486. doi: 10.3390/dj14080486 (PMC13510924; doi:10.3390/dj14080486)
Supplement: Supplementary file 1 [file dentistry-14-00486-s001.zip › Supplementary_Material_S1.pdf]

Supplementary Material S1

Codebook and reproducible analysis code

This file accompanies the manuscript and provides the operational scoring rules, the item-level answer key mapped to the analytical dataset, and the complete statistical analysis script. Together with Supplementary Material S1 (questionnaire and response distributions), it allows the composite and subscale knowledge scores and all regression models to be reproduced from the de-identified response file.

S1.1 Scoring rules

Each of the 63 knowledge items was scored dichotomously. A response was scored correct (1) when it matched the answer defined a priori from the scientific literature (Table S1.1), and incorrect (0) otherwise. Consistent with the main text, "don't know" responses, blank responses and any free-text response were scored as incorrect. The response scored as correct for each item is listed in the "Coded as" column of Table S1.1. The composite score is the percentage of correct responses across all 63 items; the three subscale scores are computed identically over their respective items (general STIs, 40 items; HPV/vaccine, 14 items; HIV/AIDS, 9 items).

Table S1.1 Item-level answer key and subscale mapping

No.	Knowledge item	Subscale	Correct	Coded as
1	HPV is a sexually transmitted virus	HPV/vaccine	True	correct = '1'
2	HPV infection is preventable	HPV/vaccine	True	correct = '1'
3	HPV infection occurs only in females	HPV/vaccine	False	correct = '0'
4	HPV can cause cancer of the female genital organs	HPV/vaccine	True	correct = '1'
5	HPV can cause cancer	HPV/vaccine	True	correct = '1'
6	HPV can also cause cancer of the mouth and throat (oropharynx)	HPV/vaccine	True	correct = '1'
7	HPV infection can cause cervical cancer	HPV/vaccine	True	correct = '1'
8	HPV can cause genital warts (condylomas)	HPV/vaccine	True	correct = '1'
9	Most HPV infections clear spontaneously	HPV/vaccine	True	correct = '1'
10	HPV can be treated with antibiotics	HPV/vaccine	False	correct = '0'
11	HIV/AIDS is transmitted through sexual intercourse	HIV/AIDS	True	correct = '1'
12	HIV/AIDS is transmitted through blood transfusion	HIV/AIDS	True	correct = '1'
13	HIV/AIDS is transmitted through needle sharing	HIV/AIDS	True	correct = '1'
14	HIV/AIDS is transmitted through food sharing	HIV/AIDS	False	correct = '1'
15	HIV/AIDS is transmitted through clothing sharing	HIV/AIDS	False	correct = '0'
16	HIV/AIDS is transmitted from mother to fetus	HIV/AIDS	True	correct = '1'
17	HIV/AIDS is transmitted through kissing (saliva)	HIV/AIDS	False	correct = '0'
18	Gonorrhoea is a sexually transmitted infection	General STIs	True	correct = '1'
19	Syphilis is a sexually transmitted infection	General STIs	True	correct = '1'
20	Genital herpes (herpes simplex) is a sexually transmitted infection	General STIs	True	correct = '1'
21	Tuberculosis is a sexually transmitted infection	General STIs	False	correct = '0'
22	Trichomoniasis is a sexually transmitted infection	General STIs	True	correct = '1'
23	Asthma is a sexually transmitted infection	General STIs	False	correct = '0'
24	HIV/AIDS is a sexually transmitted infection	General STIs	True	correct = '1'
25	Chlamydia is a sexually transmitted infection	General STIs	True	correct = '1'
26	Hepatitis B is a sexually transmitted infection	General STIs	True	correct = '1'

27	Hepatitis C is a sexually transmitted infection	General STIs	True	correct = '1'
28	STIs can be caused by bacteria	General STIs	True	correct = '1'
29	STIs can be caused by viruses	General STIs	True	correct = '1'
30	STIs can be caused by parasites	General STIs	True	correct = '1'
31	STIs can be caused by poor female hygiene	General STIs	False	correct = '0'
32	STIs can be caused by poor male hygiene	General STIs	False	correct = '0'
33	STIs can be caused by use of unclean water	General STIs	False	correct = '0'
34	STIs can be caused by having sex during menstruation	General STIs	False	correct = '0'
35	STIs are transmitted through sexual intercourse	General STIs	True	correct = '1'
36	STIs are transmitted through contact with body fluids	General STIs	True	correct = '1'
37	STIs are transmitted through oral sex	General STIs	True	correct = '1'
38	STIs are transmitted from mother to child	General STIs	True	correct = '1'
39	STIs are transmitted through coughing and colds	General STIs	False	correct = '0'
40	STIs are transmitted through ordinary social contact	General STIs	False	correct = '0'
41	STIs are transmitted through kissing	General STIs	False	correct = '0'
42	Genital ulcers can be a symptom of an STI	General STIs	True	correct = '1'
43	Genital discharge can be a symptom of an STI	General STIs	True	correct = '1'
44	Genital itching can be a symptom of an STI	General STIs	True	correct = '1'
45	Lower abdominal pain can be a symptom of an STI	General STIs	True	correct = '1'
46	Painless sores on the genitals can be a symptom of an STI	General STIs	True	correct = '1'
47	Infertility can be a consequence of an STI	General STIs	True	correct = '1'
48	Blindness can be a consequence of an STI	General STIs	True	correct = '1'
49	Headache can be a symptom of an STI	General STIs	False	correct = '0'
50	Haematuria (blood in the urine) can be a symptom of an STI	General STIs	True	correct = '1'
51	Impotence can be a consequence of an STI	General STIs	False	correct = '0'
52	Many people with an STI have no symptoms	General STIs	True	correct = '1'
53	Using oral contraceptives reduces the risk of acquiring an STI	General STIs	False	correct = '1'
54	Using condoms during intercourse reduces the risk of acquiring an STI	General STIs	True	correct = '1'
55	Having sex with multiple partners increases the risk of acquiring an STI	General STIs	True	correct = '1'
56	Untreated STIs can lead to death	General STIs	True	correct = '1'
57	Condoms protect against STIs 100% of the time	General STIs	False	correct = '1'
58	AIDS causes HIV	HIV/AIDS	False	correct = '0'
59	HIV causes AIDS	HIV/AIDS	True	correct = '1'
60	The HPV vaccine is recommended only for females	HPV/vaccine	False	correct = '0'
61	After the first sexual intercourse it is too late to be vaccinated against HPV	HPV/vaccine	False	correct = '0'
62	The HPV vaccine is now also recommended for males	HPV/vaccine	True	correct = '1'
63	Vaccination is the most effective method to prevent HPV infection	HPV/vaccine	True	correct = '1'

S1.2 Statistical analysis code (Python)

The script below reproduces the scoring, the composite and subscale scores, the adequacy thresholds, the primary linear model, the secondary logistic model with multicollinearity diagnostics, the Firth penalized-likelihood model for the rare ≥ 60% outcome, and the ≥50% sensitivity model. Requires Python 3 with pandas, numpy, statsmodels and scipy.

```
"""
Supplementary Material S2 - Reproducible scoring and analysis code
KAP study on STIs, HPV and HPV-related oropharyngeal cancer (Italian adolescents).

Input : de-identified response file (one row per participant; the header row
       holds the questionnaire item text). Requires: pandas, numpy, statsmodels, scipy.
Output : composite and subscale knowledge scores, adequacy thresholds,
        multivariable linear and logistic models, Firth penalized model,
        and multicollinearity / sensitivity diagnostics.
"""
import pandas as pd, numpy as np
import statsmodels.api as sm, statsmodels.formula.api as smf
from statsmodels.stats.outliers_influence import variance_inflation_factor
from scipy.stats import norm

# ----------------------------------------------------------------------
# 1. Load. Row 0 = item texts; participant data start at row 1.
# ----------------------------------------------------------------------
raw = pd.read_excel("responses.xlsx", header=None)
df  = raw.iloc[1:].reset_index(drop=True)
df.columns = range(df.shape[1])
S = df.astype(str).apply(lambda c: c.str.strip())

# ----------------------------------------------------------------------
# 2. Answer key for the 63 knowledge items (dataset column -> correct code)
#    The correct response for each item is defined in Table S1.1;
#    correct responses score 1 and all others (including 'don't know') score 0.
#    Any other response ('non lo so' / blank / free text) scores 0.
# ----------------------------------------------------------------------
KEY = {18:'1',19:'1',20:'0',21:'1',22:'1',23:'1',25:'1',26:'1',27:'1',28:'0',
      33:'1',34:'1',35:'1',36:'1',37:'0',38:'1',39:'0',
      47:'1',48:'1',49:'1',50:'0',51:'1',52:'0',53:'1',54:'1',55:'1',56:'1',
      57:'1',58:'1',59:'1',60:'0',61:'0',62:'0',63:'0',
      64:'1',65:'1',66:'1',67:'1',68:'0',69:'0',70:'0',
      71:'1',72:'1',73:'1',74:'1',75:'1',76:'1',77:'1',78:'0',79:'1',80:'0',
      81:'1',82:'1',83:'1',84:'1',85:'1',86:'1',31:'0',32:'1',88:'0',89:'0',90:'1',94:'1'}

# Subscale membership (see codebook, Table S1.1)
HPV = [18,19,20,21,22,23,25,26,27,28,88,89,90,94]     # HPV / vaccine  (14)
HIV = [31,32,33,34,35,36,37,38,39]                    # HIV / AIDS     (9)
STI = [c for c in KEY if c not in HPV and c not in HIV]  # general STIs (40)

def pct_correct(cols):
   M = np.column_stack([(S[c] == KEY[c]).astype(int).values for c in cols])
   return M.mean(axis=1) * 100

score      = pct_correct(list(KEY))   # composite (63 items)
score_hpv  = pct_correct(HPV)
score_hiv  = pct_correct(HIV)
score_sti  = pct_correct(STI)
```

```
# ----------------------------------------------------------------------
# 3. Analytical sample and predictors
# ----------------------------------------------------------------------
d = pd.DataFrame({'score': score})
d['female']     = (S[2] == 'Femmina').astype(float)
d['age']        = pd.to_numeric(S[3], errors='coerce')
d['sexexp']     = S[7].map({'si': 1.0, 'No': 0.0})
d['highschool'] = (S[6] == 'liceo').astype(float)
d['sexedu']     = S[14].map({'si': 1.0, 'no': 0.0})
edu = {'conseguimento diploma scuola elemantare': 1, 'conseguimento diploma terza media': 2,
       'conseguimento diploma di maturità': 3, 'conseguimento titolo di laurea': 4}
d['paredu'] = pd.concat([S[4].map(edu), S[5].map(edu)], axis=1).max(axis=1)
d = d[S[1] == 'si'].copy()          # keep authorized questionnaires
for t in (50, 60, 70):
    d[f'adeq{t}'] = (d['score'] >= t).astype(int)

print("N =", len(d), "| mean =", round(d.score.mean(), 1), "| SD =", round(d.score.std(), 1))
print("Subscales -> STI %.1f  HPV %.1f  HIV %.1f" %
      (score_sti.mean(), score_hpv.mean(), score_hiv.mean()))
for t in (50, 60, 70):
    print("  adequacy >=%d%%: %.1f%% (n=%d)" % (t, d[f'adeq{t}'].mean()*100, d[f'adeq{t}'].sum()))

# ----------------------------------------------------------------------
# 4. Primary outcome: multivariable linear regression (continuous score)
# ----------------------------------------------------------------------
lin = smf.ols('score ~ female+age+sexexp+highschool+sexedu+paredu', data=d).fit()
print("\nLinear model R2 =", round(lin.rsquared, 3))
print(lin.summary2().tables[1][['Coef.', '[0.025', '0.975]', 'P>|t|']].round(3))

# ----------------------------------------------------------------------
# 5. Secondary outcome: logistic (>=60%) + diagnostics
# ----------------------------------------------------------------------
dd = d.dropna(subset=['female','age','sexexp','highschool','sexedu','paredu']).copy()
log = smf.logit('adeq60 ~ female+age+sexexp+highschool+sexedu+paredu', data=dd).fit(disp=0)

X = sm.add_constant(dd[['female','age','sexexp','highschool','sexedu','paredu']].astype(float))
vif = {c: round(variance_inflation_factor(X.values, i), 2)
       for i, c in enumerate(X.columns) if c != 'const'}
print("\nLogistic >=60%: events =", int(dd.adeq60.sum()), "| VIF =", vif)

# ----------------------------------------------------------------------
# 6. Firth penalized logistic (rare-outcome robustness)
# ----------------------------------------------------------------------
def firth(X, y, itmax=500, tol=1e-9):
    b = np.zeros(X.shape[1])
    for _ in range(itmax):
        p = 1/(1+np.exp(-(X@b))); W = p*(1-p)
        Iinv = np.linalg.pinv((X.T*W)@X); rW = np.sqrt(W)
        h = np.diag((rW[:, None]*X)@Iinv@(X.T*rW))
        step = Iinv@(X.T@(y-p+h*(0.5-p))); b = b+step
        if np.max(np.abs(step)) < tol: break
    return b, np.sqrt(np.diag(Iinv))

Xf = np.column_stack([np.ones(len(dd))] +
```

```
                  [dd[c].values.astype(float) for c in
                   ['female','age','sexexp','highschool','sexedu','paredu']])
bf, sef = firth(Xf, dd.adeq60.values.astype(float))
pf = 2*(1-norm.cdf(np.abs(bf/sef)))
print("Firth OR (female, age, sexexp) =",
     [round(np.exp(bf[i]), 2) for i in (1, 2, 3)])

# ----------------------------------------------------------------------
# 7. Sensitivity: logistic at >=50% (more events)
# ----------------------------------------------------------------------
log50 = smf.logit('adeq50 ~ female+age+sexexp+highschool+sexedu+paredu', data=dd).fit(disp=0)
print("Logistic >=50%: events =", int(dd.adeq50.sum()),
     "| female OR =", round(np.exp(log50.params['female']), 2),
     "| age OR =", round(np.exp(log50.params['age']), 2))
```
