# Supplementary material for: Knowledge, Attitudes, and Practices (KAP) Regarding Sexually Transmitted Infections, Human Papillomavirus, and HPV-Related Oropharyngeal Cancer Among Italian Adolescents: A Cross-Sectional Study
Source: Dent J (Basel). 2026 Aug 5;14(8):486. doi: 10.3390/dj14080486 (PMC13510924; doi:10.3390/dj14080486)
Supplement: Supplementary file 1 [file dentistry-14-00486-s001.zip › Supplementary_Material_S2.pdf]

Supplementary Material S2. Questionnaire development and content validation

Source instruments:

1. Bayramoğlu Tepe, N., & Ozcorekci, O. (2020). Knowledge about the human papillomavirus among high school and university students a comprehensive questionnaire study from Southeast Turkey. *The journal of obstetrics and gynaecology research*, *46*(6), 907–916. https://doi.org/10.1111/jog.14247
2. Visalli, G., Picerno, I., Vita, G., Spataro, P., & Bertuccio, M. P. (2014). Knowledge of sexually transmitted infections among younger subjects of the city of Messina (Sicily). *Journal of preventive medicine and hygiene*, *55*(1), 17–22.
3. Bocquier, A., Branchereau, M., Gauchet, A., Bonnay, S., Simon, M., Ecollan, M., Chevreul, K., Mueller, J. E., Gagneux-Brunon, A., Thilly, N., & PrevHPV Study Group (2023). Promoting HPV vaccination at school: a mixed methods study exploring knowledge, beliefs and attitudes of French school staff. *BMC public health*, *23*(1), 486. https://doi.org/10.1186/s12889-023-15342-2
4. Biyazin, T., Yilma, A., Yetwale, A., Fenta, B., & Dagnaw, Y. (2022). Knowledge and attitude about human papillomavirus vaccine among female high school students at Jimma town, Ethiopia. *Human vaccines & immunotherapeutics*, *18*(1), 2036522. https://doi.org/10.1080/21645515.2022.2036522
5. Mutabazi, S., Esaete, J., & Kansiime, E. (2023). Education level, students' knowledge and attitude towards STIs in selected secondary schools in Kisoro municipality, Western Uganda. *Social Sciences & Humanities Open*, *7*(1), 100475. https://doi.org/10.1016/j.ssaho.2023.100475

Part 1 (Item-by-item source mapping)

#	Questionnaire item	Class.
**Section A. General knowledge of HPV**		
1	HPV is a sexually transmitted virus	Included
2	HPV infection is preventable	Included (adapted)
3	HPV infection occurs only in females	Included (adapted)
4	HPV can cause cancer of the female genital organs	Included (adapted)
5	HPV can cause cancer	Included (adapted)
6	HPV can also cause cancer of the mouth and throat (oropharynx)	Included (adapted)
7	HPV infection can cause cervical cancer	Included
8	HPV can cause genital warts (condylomas)	Included
9	Most HPV infections clear spontaneously	Included (adapted)
10	HPV can be treated with antibiotics	Included
**Section B. Modes of HIV/AIDS transmission**		
11	HIV/AIDS is transmitted through sexual intercourse	Included (adapted)
12	HIV/AIDS is transmitted through blood transfusion	Included (adapted)
13	HIV/AIDS is transmitted through needle sharing	Included (adapted)
14	HIV/AIDS is transmitted through food sharing	Included (adapted)
15	HIV/AIDS is transmitted through clothing sharing	Included (adapted)
16	HIV/AIDS is transmitted through mother to fetus	Included (adapted)
17	HIV/AIDS is transmitted through kissing (saliva)	Included (adapted)

**Section C. Recognition of STIs**		
18	Gonorrhoea is a sexually transmitted infection	Included
19	Syphilis is a sexually transmitted infection	Included
20	Genital herpes is a sexually transmitted infection	Included
21	Tuberculosis is a sexually transmitted infection	Included
22	Trichomoniasis is a sexually transmitted infection	Included
23	Asthma is a sexually transmitted infection	Included
24	HIV/AIDS is a sexually transmitted infection	Included
25	Chlamydia is a sexually transmitted infection	Included
26	Hepatitis B is a sexually transmitted infection	Included
27	Hepatitis C is a sexually transmitted infection	Included
**Section D. Causes of STIs**		
28	STIs can be caused by bacteria	Included
29	STIs can be caused by viruses	Included (adapted)
30	STIs can be caused by parasites	Included (adapted)
31	STIs can be caused by poor female hygiene	Included (adapted)
32	STIs can be caused by poor male hygiene	Included (adapted)
33	STIs can be caused by use of unclean water	Included (adapted)
34	STIs can be caused by having sex during menstruation	Included (adapted)
**Section E. Modes of STI transmission**		
35	STIs are transmitted through sexual intercourse	Included (adapted)
36	STIs are transmitted through contact with body fluids	Included
37	STIs are transmitted through oral sex	Included (adapted)
38	STIs are transmitted through mother to child	Included (adapted)
39	STIs are transmitted through coughing and colds	Included (adapted)
40	STIs are transmitted through ordinary social contact	Included (adapted)
41	STIs are transmitted through kissing	Included (adapted)
**Section F. Symptoms and consequences of STIs**		
42	Genital ulcers can be a symptom	Included (adapted)
43	Genital discharge can be a symptom	Included (adapted)
44	Genital itching can be a symptom	Included (adapted)
45	Lower abdominal pain can be a symptom	Included
46	Painless sores can be a symptom	Included
47	Infertility can be a consequence	Included
48	Blindness can be a consequence	Included (adapted)
49	Headache can be a symptom	Included
50	Haematuria can be a symptom	Included (adapted)
51	Impotence can be a consequence	Included (adapted)
**Section G. Prevention, HIV/AIDS and HPV vaccination**		
52	Many people with an STI have no symptoms	Included (adapted)
53	Using oral contraceptives reduces STI risk	Included (adapted)

54	Using condoms reduces STI risk	Included (adapted)
55	Multiple partners increase STI risk	Included (adapted)
56	Untreated STIs can lead to death	Included (adapted)
57	Condoms protect against STIs 100% of the time	Included (adapted)
58	AIDS causes HIV	Included
59	HIV causes AIDS	Included
60	The HPV vaccine is recommended only for females	Included (adapted)
61	After first intercourse it is too late to be vaccinated	Included (adapted)
62	The HPV vaccine is now also recommended for males	Included (adapted)
63	Vaccination is the most effective method to prevent HPV	Included (adapted)

Part 1. Candidate item pool and selection (Stage 1)

All items screened from the source instruments. Items were removed before expert review when they duplicated an item already included (Excluded - redundant), when their original response format was incompatible with the true/false (T/F) format (Excluded - format; e.g., self-rated, multiple-choice or open-ended), or when, although relevant, they were set aside to limit questionnaire length and respondent burden (Excluded - scope/length).

Candidate item	Format	Status
HPV infections are only encountered in females	T/F	Included
After HPV transmission, symptoms take years to manifest	T/F	Excluded – scope/length
The only type of cancer caused by HPV is cervical cancer	T/F	Included
The HPV vaccine is only recommended for females	T/F	Included
The HPV vaccine has serious side effects	T/F	Excluded – scope/length
All sexually active individuals can become infected with HPV	T/F	Excluded – scope/length
Condoms offer complete protection against HPV	T/F	Included
It is possible to know who will develop cancer from HPV	T/F	Excluded – scope/length
HPV infection is a prevalent infection	T/F	Excluded – scope/length
HPV is a sexually transmitted virus	T/F	Included
HPV infection may cause genital warts	T/F	Included
HPV infection may cause cervical cancer	T/F	Included
The HPV vaccine protects against all types of HPV	T/F	Excluded – scope/length
Smear tests are not required after the HPV vaccine	T/F	Excluded – scope/length
HPV infection is a preventable infection	T/F	Included
HPV may affect both females and males	T/F	Excluded – redundant
Most HPV infections spontaneously regress	T/F	Included
The current HPV vaccine prevents 70% of cervical cancers	T/F	Excluded – scope/length
Cervical cancer is a severe disease	T/F	Excluded – scope/length
Cervical cancer is a preventable disease	T/F	Excluded – scope/length
HPV vaccine also effective against genital warts	T/F	Excluded – scope/length
Women infected with HPV do not need vaccination	T/F	Excluded – scope/length
Types of HPV causing warts and cancer are the same	T/F	Excluded – scope/length
An abnormal smear may indicate HPV infection	T/F	Excluded – scope/length
Most individuals infected with HPV are asymptomatic	T/F	Excluded – scope/length
Recovery is not an option in the case of HPV	T/F	Excluded – scope/length
The number of men infected with HPV is quite low	T/F	Excluded – scope/length
HPV infection leads to cervical cancer in all cases	T/F	Excluded – scope/length
HPV infection can be treated with antibiotics	T/F	Included
Individuals with HPV must undergo smear tests more often	T/F	Excluded – scope/length
What STIs do you know? (list all)	Open	Excluded – format

How is the infection transmitted? [sex, body fluids, oral sex, petting, kiss, coughing, mother-child, social contact]	Checklist	Included
Which clinical manifestations? [genital lesions, infertility, brain injury, blindness, AIDS, headache, diarrhoea, haematuria, powerlessness]	Checklist	Included
How can you protect yourself from STIs? (open)	Open	Excluded – format
HIV is? [bacterium / virus / fungus / disease]	MC	Excluded – scope/length
AIDS is? [virus / infectious / hereditary / opportunistic]	MC	Excluded – scope/length
How is HIV transmitted? [kiss, blood, transfusion, syringes, living with HIV+, sex, pregnancy]	Checklist	Excluded – redundant
Which body fluids contain HIV? [saliva, sperm, sweat, precoital, blood, tears]	Checklist	Excluded – scope/length
Which is correct: AIDS causes HIV / HIV causes AIDS	MC	Included
Who is HIV-positive? [AIDS patient / antibodies / thin & worn / risk behaviour]	MC	Excluded – format
If you are HIV-positive, whom would you tell? [no-one / friend / doctor / partner / family / everyone]	Checklist	Included
Is it necessary to isolate HIV-positive people at school?	Y/N	Excluded – scope/length
Which is true? [AIDS cured / you die / vaccine exists / not in Italy / only developing]	MC	Excluded – scope/length
Condom-use self-recognition [I use / always / never]	MC	Excluded – scope/length
Can you diagnose HIV infection?	Y/N	Excluded – scope/length
Where can HIV diagnostic tests be done?	MC	Excluded – scope/length
Do HIV test results remain secret?	Y/N	Excluded – scope/length
Will a person with HIV develop AIDS?	MC	Excluded – scope/length
Main source of information on AIDS/STIs	Checklist	Included
Do you consider your knowledge sufficient?	MC	Included
From what source do you expect more information?	MC	Excluded – redundant
Gonorrhoea / Syphilis / Genital herpes / Trichomoniasis / Tuberculosis / Asthma / HIV / Chlamydia / Hepatitis B / Hepatitis C	Checklist	Included
Have you ever heard of STIs?	Y/N	Included
STIs are diseases transmitted through sex	Y/N	Excluded – redundant
STIs affect the sex organs mainly	Y/N	Excluded – redundant
Causes: Bacteria / Virus / Parasites / Bad hygiene (F) / Bad hygiene (M) / Unclean water / Sex during menstruation	Checklist	Included
Ulcers in the sexual organs	Y/N	Included
Pain while passing urine	Y/N	Excluded – scope/length
Swelling of sexual organs / failure to urinate	Y/N	Excluded – scope/length
Discharge from the penis/vagina	Y/N	Included
Itching around the vagina/tip of the penis	Y/N	Included
Lower abdominal pain	Y/N	Included
Painless sores on the sexual organs	Y/N	Included

Some people with STIs have no symptoms	Y/N	Included
Routes of HIV: sexual intercourse / blood transfusion / sharing needles / sharing food / sharing clothes / mother-to-child / kissing	Checklist	Included
Effect of STI: infertility	Y/N	Included
Effect of STI: cervical cancer	Y/N	Excluded – scope/length
Effect of STI: body weakness	Y/N	Excluded – redundant
Effect of STI: ectopic pregnancy	Y/N	Excluded – scope/length
Effect of STI: stillbirth	Y/N	Excluded – scope/length
Use of contraceptive pills reduces STI risk	Y/N	Included
Use of condoms reduces STI risk	Y/N	Included
Alcohol intake increases STI risk	Y/N	Excluded – scope/length
Having sex with many people increases STI risk	Y/N	Included
Sexual abstinence is the most effective means	Y/N	Excluded – scope/length
Attitude: condoms protect against STIs and HIV	3-pt	Excluded – scope/length
Attitude: not necessary to use condoms during sex	3-pt	Excluded – scope/length
Attitude: if both infected, no need for a condom	3-pt	Excluded – scope/length
Attitude: numerous partners play no role in transmission	3-pt	Excluded – redundant
Attitude: banning prostitution can control spread	3-pt	Excluded – scope/length
Attitude: screening for STIs/HIV is good	3-pt	Excluded – scope/length
Attitude: STIs are not dangerous because curable	3-pt	Excluded – scope/length
Attitude: STIs can cause death if left untreated	3-pt	Included
Attitude: I am worried about getting STIs/HIV	3-pt	Included
Attitude: the STI problem is something I have not thought about	3-pt	Excluded – redundant
Attitude: unprotected sex → most concerned about HIV	3-pt	Excluded – scope/length
Attitude: unprotected sex → most concerned about other STIs	3-pt	Excluded – scope/length
Attitude: unprotected sex → most concerned about pregnancy	3-pt	Included
Attitude: some STIs are not curable	3-pt	Excluded – redundant
Attitude: if I notice symptoms I should seek treatment	3-pt	Excluded – scope/length
Attitude: if partner has symptoms, I would advise treatment	3-pt	Included
Attitude: sexually active people get STIs more often	3-pt	Excluded – scope/length
HPV is a sexually transmitted virus	T/F	Excluded – redundant
HPV also concerns boys and men	T/F	Excluded – redundant
Different types of HPV exist; only some cause cancers	T/F	Included
HPV infection is always symptomatic	T/F	Excluded – scope/length
More than half of people are infected during life	T/F	Excluded – scope/length
HPV causes genital warts	T/F	Excluded – redundant
Cervical cancer is due to a persistent HPV infection	T/F	Excluded – redundant
HPV can also cause oral cancers	T/F	Included
Most HPV infections are eliminated spontaneously	T/F	Excluded – redundant
There is no antiviral treatment against HPV	T/F	Excluded – redundant
There is a vaccine against HPV	T/F	Excluded – scope/length

Vaccinating girls leads to earlier sexual activity	T/F	Excluded – scope/length
Cervical screening still recommended among vaccinated women	T/F	Excluded – scope/length
HPV vaccine protects against cancer-causing virus	T/F	Excluded – redundant
HPV vaccine prevents precancerous cervical lesions	T/F	Excluded – scope/length
After first intercourse it is too late to be vaccinated	T/F	Included
HPV vaccine is recommended for heterosexual boys	T/F	Included
HPV vaccine is responsible for many side effects	T/F	Excluded – redundant
HPV vaccine recommended for MSM/bisexual boys ≤26y	T/F	Excluded – scope/length
HPV vaccine can eliminate an existing infection	T/F	Excluded – scope/length
HPV vaccine protects against genital warts	T/F	Excluded – scope/length
A condom protects against HPV infection	T/F	Excluded – scope/length
I feel at risk of HPV, I will take the vaccine	3-pt	Excluded – scope/length
Being infected with HPV is very deadly	3-pt	Excluded – scope/length
The vaccine will keep me safe and healthy	3-pt	Included
My parents must decide whether I take the vaccine	3-pt	Included
The cost of the vaccine discourages me	3-pt	Included
The safety/protection of the vaccine encourages me	3-pt	Included
I would use the vaccine if free at school	3-pt	Excluded – scope/length
I feel embarrassed to get vaccinated	3-pt	Excluded – scope/length
I do not know much about the vaccine, so I will not take it	3-pt	Excluded – scope/length
Have you heard about the HPV vaccine?	Y/N	Excluded – redundant
HPV vaccine is offered free to school girls of a given age	Y/N	Included
HPV vaccine is given in 2–3 doses	Y/N	Included
HPV vaccine is most effective before sexual activity	Y/N	Excluded – redundant
HPV vaccination is allowed in all religions	Y/N	Excluded – scope/length
HPV vaccine prevents genital warts & cervical cancer	Y/N	Excluded – redundant
The HPV vaccine is accessible to men	Y/N	Excluded – redundant
Ideal age range to receive the HPV vaccine	MC	Included
Have you ever heard about HPV?	Y/N	Excluded – format
HPV can lead to cervical cancer	Y/N	Excluded – redundant
HPV can lead to other forms of cancer	Y/N	Excluded – redundant
HPV can lead to condyloma	Y/N	Excluded – redundant
HPV can be transmitted through sexual intercourse	Y/N	Excluded – redundant
Women can contract HPV	Y/N	Excluded – redundant
Men can contract HPV	Y/N	Excluded – redundant
There is a vaccine against HPV	Y/N	Excluded – scope/length
Do you know what HPV is?	Self-rated	Excluded – format
Do you know how HPV is transmitted?	Self-rated	Excluded – format
Do you know how HPV is prevented?	Self-rated	Excluded – format
Have you ever been concerned with HPV?	Self-rated	Excluded – format
Have you sought health care due to HPV concerns?	Self-rated	Excluded – format

Do you know what a Pap smear is?	Self-rated	Excluded – format
Do you know what the cervix is?	Self-rated	Excluded – format
Do you know what cancer is?	Self-rated	Excluded – format
Do you know what cervical cancer is?	Self-rated	Excluded – format
Do you know what an STD is?	Self-rated	Excluded – format
Do you know how to prevent STDs?	Self-rated	Excluded – format
Do you know what a condom is?	Self-rated	Included
Do you know someone who has had an STD?	Self-rated	Excluded – format
Have you heard of cervical cancer?	Y/N	Excluded – format
Do you think you are at risk of cervical cancer?	Y/N	Excluded – format
Have you heard of HPV?	Y/N	Excluded – format
Have you heard of HPV vaccines?	Y/N	Excluded – format
HPV vaccine can prevent cervical/anal cancer/genital warts	Y/N	Excluded – redundant
Prior consultation regarding HPV vaccines	Y/N	Excluded – format
Willing to be vaccinated against HPV	Y/N	Included
Main cause of cervical cancer? (MC)	MC	Excluded – format
Transmission route of HPV? (MC)	MC	Excluded – redundant
Most effective method of preventing HPV? (MC)	MC	Included
Best age to get vaccinated? (MC)	MC	Excluded – redundant
Willing to be vaccinated if available	Y/N	Excluded – redundant

Part 2. Content validity indices (Stage 2)

Five experts independently rated each item for relevance on a four-point scale. I-CVI = proportion rating 3–4; κ* = chance-corrected agreement; items retained at I-CVI ≥ 0.78 (Lynn 1986).

Panel: three oral-medicine physicians (two with HPV-KAP expertise), one clinically experienced dentist and one educationalist; an infectious-disease specialist was additionally consulted (qualitative review of STI/HIV items only.

ID	Item	I-CVI	κ*	Decision
**Knowledge**				
1	HPV is a sexually transmitted virus	1.00	1.00	Retained
2	HPV infection is preventable	1.00	1.00	Retained
3	HPV infection occurs only in females	1.00	1.00	Retained
4	HPV can cause cancer of the female genital organs	0.80	0.76	Retained
5	HPV can cause cancer	1.00	1.00	Retained
6	HPV can also cause cancer of the mouth and throat (oropharynx)	1.00	1.00	Retained
7	HPV infection can cause cervical cancer	1.00	1.00	Retained
8	HPV can cause genital warts (condylomas)	1.00	1.00	Retained
9	Most HPV infections clear spontaneously	1.00	1.00	Retained
10	HPV can be treated with antibiotics	0.80	0.76	Retained
11	HIV/AIDS transmitted through sexual intercourse	1.00	1.00	Retained
12	HIV/AIDS through blood transfusion	1.00	1.00	Retained
13	HIV/AIDS through needle sharing	1.00	1.00	Retained
14	HIV/AIDS through food sharing	1.00	1.00	Retained
15	HIV/AIDS through clothing sharing	1.00	1.00	Retained
16	HIV/AIDS from mother to fetus	1.00	1.00	Retained
17	HIV/AIDS through kissing (saliva)	1.00	1.00	Retained
18	Gonorrhoea is an STI	1.00	1.00	Retained
19	Syphilis is an STI	1.00	1.00	Retained
20	Genital herpes is an STI	1.00	1.00	Retained
21	Tuberculosis is an STI	1.00	1.00	Retained
22	Trichomoniasis is an STI	1.00	1.00	Retained
23	Asthma is an STI	1.00	1.00	Retained
24	HIV/AIDS is an STI	1.00	1.00	Retained
25	Chlamydia is an STI	1.00	1.00	Retained
26	Hepatitis B is an STI	1.00	1.00	Retained
27	Hepatitis C is an STI	1.00	1.00	Retained
28	STIs caused by bacteria	1.00	1.00	Retained
29	STIs caused by viruses	1.00	1.00	Retained
30	STIs caused by parasites	1.00	1.00	Retained
31	STIs caused by poor female hygiene	1.00	1.00	Retained

32	STIs caused by poor male hygiene	1.00	1.00	Retained
33	STIs caused by unclean water	0.80	0.76	Retained
34	STIs caused by sex during menstruation	1.00	1.00	Retained
35	STIs transmitted through sexual intercourse	1.00	1.00	Retained
36	STIs through contact with body fluids	1.00	1.00	Retained
37	STIs through oral sex	1.00	1.00	Retained
38	STIs from mother to child	1.00	1.00	Retained
39	STIs through coughing and colds	0.80	0.76	Retained
40	STIs through ordinary social contact	1.00	1.00	Retained
41	STIs through kissing	1.00	1.00	Retained
42	Genital ulcers can be a symptom	1.00	1.00	Retained
43	Genital discharge can be a symptom	1.00	1.00	Retained
44	Genital itching can be a symptom	1.00	1.00	Retained
45	Lower abdominal pain can be a symptom	1.00	1.00	Retained
46	Painless sores can be a symptom	0.80	0.76	Retained
47	Infertility can be a consequence	1.00	1.00	Retained
48	Blindness can be a consequence	1.00	1.00	Retained
49	Headache can be a symptom	1.00	1.00	Retained
50	Haematuria can be a symptom	1.00	1.00	Retained
51	Impotence can be a consequence	1.00	1.00	Retained
52	Many people with an STI have no symptoms	1.00	1.00	Retained
53	Oral contraceptives reduce STI risk	1.00	1.00	Retained
54	Condoms reduce STI risk	1.00	1.00	Retained
55	Multiple partners increase STI risk	1.00	1.00	Retained
56	Untreated STIs can lead to death	1.00	1.00	Retained
57	Condoms protect against STIs 100%	1.00	1.00	Retained
58	AIDS causes HIV	1.00	1.00	Retained
59	HIV causes AIDS	1.00	1.00	Retained
60	HPV vaccine only for females	1.00	1.00	Retained
61	After first intercourse too late to be vaccinated	1.00	1.00	Retained
62	HPV vaccine also for males	1.00	1.00	Retained
63	Vaccination is the most effective method to prevent HPV	1.00	1.00	Retained
**Behaviour**				
B1	Have you ever had sexual intercourse?	1.00	1.00	Retained
B2	Are you currently sexually active / in a relationship?	1.00	1.00	Retained
B3	Do you know what a condom is?	1.00	1.00	Retained
B4	Which protective measures do you usually use during intercourse?	1.00	1.00	Retained
B5	Would you use a condom with a new sexual partner?	1.00	1.00	Retained
B6	Would you use a condom if you/your partner use oral contraceptives?	1.00	1.00	Retained
B7	Do you find it embarrassing to buy condoms?	1.00	1.00	Retained
B8	Have you received sex education at school?	1.00	1.00	Retained

B9	Have you ever heard of STIs?	1.00	1.00	Retained
B10	With unprotected sex, are you more worried about pregnancy?	1.00	1.00	Retained
**Attitude**				
A1	If HIV-positive, you would tell no one	1.00	1.00	Retained
A2	If HIV-positive, you would tell your family doctor	1.00	1.00	Retained
A3	If HIV-positive, you would tell your best friend	1.00	1.00	Retained
A4	If HIV-positive, you would tell your partner	1.00	1.00	Retained
A5	If HIV-positive, you would tell your family	1.00	1.00	Retained
A6	If HIV-positive, you would tell everyone	1.00	1.00	Retained
A7	If you contracted an STI, would you inform your partner?	1.00	1.00	Retained
A8	Do you know the ideal age range for the HPV vaccine?	1.00	1.00	Retained
A9	Are you aware the vaccine is free for girls of a given age?	1.00	1.00	Retained
A10	Are you aware the HPV vaccine is given in 2–3 doses?	1.00	1.00	Retained
A11	STIs are not something I worry about	1.00	1.00	Retained
A12	I would like to receive the HPV vaccine myself	1.00	1.00	Retained
A13	I have already received the HPV vaccine	1.00	1.00	Retained
A14	I have completed all doses of the HPV vaccine	1.00	1.00	Retained
A15	The HPV vaccine allows us to stay safe and healthy	1.00	1.00	Retained
A16	My parents decide whether I get the HPV vaccine	1.00	1.00	Retained
A17	The cost of the vaccine discourages me	0.80	0.76	Retained
A18	The protection the HPV vaccine gives encourages me	1.00	1.00	Retained
A19	Do you consider your knowledge of STIs satisfactory?	1.00	1.00	Retained
A20	Where did you learn about the existence of STIs?	1.00	1.00	Retained
A21	Which STI do you think is the most common?	1.00	1.00	Retained
A22	I need more information about HPV infection	1.00	1.00	Retained
A23	I need more information about the HPV vaccine	1.00	1.00	Retained
**Rated but not retained**				
N1	The current HPV vaccine prevents 70% of cervical cancers	0.60	0.42	Not retained
N2	Individuals with HPV must undergo smear tests more often	0.60	0.42	Not retained
N3	HPV vaccine recommended for MSM/bisexual boys (≤26 y)	0.60	0.42	Not retained
N4	Alcohol intake increases the risk of STIs	0.40	0.13	Not retained
