# Supplementary material for: Knowledge, Attitudes, and Practices (KAP) Regarding Sexually Transmitted Infections, Human Papillomavirus, and HPV-Related Oropharyngeal Cancer Among Italian Adolescents: A Cross-Sectional Study
Source: Dent J (Basel). 2026 Aug 5;14(8):486. doi: 10.3390/dj14080486 (PMC13510924; doi:10.3390/dj14080486)
Supplement: Supplementary file 1 [file dentistry-14-00486-s001.zip › Supplementary_Material_S3.pdf]

Supplementary Material S3

Full questionnaire and distribution of responses

This supplementary file accompanies the manuscript “A Knowledge, Attitudes and Practices (KAP) Study on Sexually Transmitted Infections, HPV and HPV-Related Oropharyngeal Cancer Among Italian Adolescents: A Cross-Sectional Survey-Based Study with Implications for the Dental Setting”. It reproduces the complete self-administered questionnaire (translated from the original Italian version). The instrument comprised four parts: socio-demographic data, sexual and preventive behaviours, a 63-item knowledge block, and attitudes, intentions and information sources. For the knowledge block, the answer scored as correct and the percentage of correct responses are reported; consistent with the main text, “don’t know” answers were scored as incorrect. For the remaining parts, which have no “correct” answer, the full distribution of responses is given. Percentages are computed on all 1,224 respondents and may not sum to exactly 100% due to rounding.

Part 1. Socio-demographic data

Q1. Sex

Response	n	%
Female	687	56.1
Male	528	43.1
Prefer not to say	9	0.7

Q2. Age (years)

Response	n	%
16	275	22.5
15	253	20.7
18	217	17.7
14	209	17.1
17	194	15.8
19	75	6.1
Other / unspecified	1	0.1

Q3. Mother's education level

Response	n	%
Upper-secondary diploma	525	42.9
University degree	363	29.7
Lower-secondary certificate	293	23.9
Primary-school certificate	24	2.0
Other / unspecified	19	1.6

Q4. Father's education level

Response	n	%
Upper-secondary diploma	533	43.5
Lower-secondary certificate	351	28.7
University degree	291	23.8
Primary-school certificate	26	2.1
Other / unspecified	23	1.9

Q5. Type of school attended

Response	n	%
General secondary school (Liceo)	643	52.5
Technical secondary school	525	42.9
Vocational secondary school	44	3.6
Vocational training center	12	1.0

Part 2. Sexual and preventive behaviours

Q1. Have you ever had sexual intercourse?

Response	n	%
No	770	62.9
Yes	454	37.1

Q2. Are you currently sexually active / in a relationship / with an occasional partner?

Response	n	%
No	860	70.3
Yes	364	29.7

Q3. Do you know what a condom is?

Response	n	%
Yes	1203	98.3
No	21	1.7

Q4. Which protective measures do you usually use during intercourse?

Response	n	%
Has never had intercourse	456	37.3
Condom	381	31.1
None	196	16.0
preservativo;pillola anticoncezionale	36	2.9
preservativo;nessuno	25	2.0
Oral contraceptive pill	15	1.2
Other / combined responses	115	9.4

Q5. Would you use a condom with a new sexual partner?

Response	n	%
Yes	1082	88.4
Don’t know	104	8.5
No	38	3.1

Q6. Would you use a condom if you or your partner use oral contraceptives?

Response	n	%
Yes	562	45.9

Response	n	%
Don’t know	375	30.6
No	287	23.4

Q7. Do you find it embarrassing to buy condoms?

Response	n	%
No	878	71.7
Yes	212	17.3
Don’t know	134	10.9

Q8. Have you received sex education at school?

Response	n	%
No	955	78.0
Yes	269	22.0

Q9. Have you ever heard of sexually transmitted infections (STIs)?

Response	n	%
Yes	1152	94.1
No	72	5.9

Q10. With unprotected sex, are you more worried about an unwanted pregnancy?

Response	n	%
Yes	946	77.3
Don’t know	159	13.0
No	119	9.7

Part 3. Knowledge items

For each item the table reports the answer defined a priori as correct, the percentage of students answering correctly, and the distribution of “yes”, “no” and “don’t know” responses.

Section A. General knowledge of HPV

#	Questionnaire item	Correct answer	Correct (%)	Yes (%)	No (%)	Don’t know (%)
1	HPV is a sexually transmitted virus	True	**68.8**	68.8	1.8	29.4
2	HPV infection is preventable	True	**50.5**	50.5	4.6	44.9
3	HPV infection occurs only in females	False	**7.3**	42.2	7.3	50.5
4	HPV can cause cancer of the female genital organs	True	**27.2**	27.2	2.7	70.1
5	HPV can cause cancer	True	**29.0**	29.0	3.2	67.8
6	HPV can also cause cancer of the mouth and throat (oropharynx)	True	**14.8**	14.8	5.7	79.5
7	HPV infection can cause cervical cancer	True	**19.8**	19.8	2.1	78.1
8	HPV can cause genital warts (condylomas)	True	**8.3**	8.3	1.7	90.0
9	Most HPV infections clear spontaneously	True	**7.5**	7.5	16.5	76.0
10	HPV can be treated with antibiotics	False	**14.0**	16.9	14.0	69.1

Section B. Modes of HIV/AIDS transmission

#	Questionnaire item	Correct answer	Correct (%)	Yes (%)	No (%)	Don't know (%)
11	HIV/AIDS is transmitted through sexual intercourse	True	**83.7**	83.7	2.8	13.6
12	HIV/AIDS is transmitted through blood transfusion	True	**53.8**	53.8	9.9	36.3
13	HIV/AIDS is transmitted through needle sharing	True	**59.3**	59.3	8.0	32.7
14	HIV/AIDS is transmitted through food sharing	False	**56.0**	11.0	56.0	33.0
15	HIV/AIDS is transmitted through clothing sharing	False	**3.7**	70.5	3.7	25.8
16	HIV/AIDS is transmitted from mother to fetus	True	**35.5**	35.5	11.9	52.6
17	HIV/AIDS is transmitted through kissing (saliva)	False	**30.0**	34.2	30.0	35.9

Section C. Recognition of sexually transmitted infections

#	Questionnaire item	Correct answer	Correct (%)	Yes (%)	No (%)	Don't know (%)
18	Gonorrhoea is a sexually transmitted infection	True	**23.1**	23.1	13.2	63.7
19	Syphilis is a sexually transmitted infection	True	**36.8**	36.8	5.8	57.4
20	Genital herpes (herpes simplex) is a sexually transmitted infection	True	**61.0**	61.0	5.9	33.1
21	Tuberculosis is a sexually transmitted infection	False	**13.0**	44.8	13.0	42.2
22	Trichomoniasis is a sexually transmitted infection	True	**8.4**	8.4	10.1	81.5
23	Asthma is a sexually transmitted infection	False	**4.4**	70.3	4.4	25.3
24	HIV/AIDS is a sexually transmitted infection	True	**79.9**	79.9	1.9	18.2
25	Chlamydia is a sexually transmitted infection	True	**28.3**	28.3	6.5	65.1
26	Hepatitis B is a sexually transmitted infection	True	**21.5**	21.5	16.9	61.6
27	Hepatitis C is a sexually transmitted infection	True	**18.5**	18.5	16.7	64.9

Section D. Causes of sexually transmitted infections

#	Questionnaire item	Correct answer	Correct (%)	Yes (%)	No (%)	Don't know (%)
28	STIs can be caused by bacteria	True	**31.9**	31.9	10.9	57.2
29	STIs can be caused by viruses	True	**47.7**	47.7	4.1	48.2
30	STIs can be caused by parasites	True	**17.5**	17.5	18.1	64.4
31	STIs can be caused by poor female hygiene	False	**36.7**	8.1	36.7	55.2
32	STIs can be caused by poor male hygiene	False	**35.9**	8.0	35.9	56.0
33	STIs can be caused by use of unclean water	False	**22.1**	15.3	22.1	62.6
34	STIs can be caused by having sex during menstruation	False	**11.3**	28.7	11.3	60.0

Section E. Modes of STI transmission

#	Questionnaire item	Correct answer	Correct (%)	Yes (%)	No (%)	Don't know (%)
35	STIs are transmitted through sexual intercourse	True	**49.4**	49.4	6.7	43.9
36	STIs are transmitted through contact with body fluids	True	**20.3**	20.3	9.9	69.9
37	STIs are transmitted through oral sex	True	**15.0**	15.0	29.7	55.3
38	STIs are transmitted from mother to child	True	**21.6**	21.6	16.2	62.3
39	STIs are transmitted through coughing and colds	False	**5.0**	43.7	5.0	51.3
40	STIs are transmitted through ordinary social contact	False	**3.8**	42.6	3.8	53.6
41	STIs are transmitted through kissing	False	**16.6**	30.5	16.6	52.9

Section F. Symptoms and consequences of STIs

#	Questionnaire item	Correct answer	Correct (%)	Yes (%)	No (%)	Don't know (%)
42	Genital ulcers can be a symptom of an STI	True	**26.2**	26.2	4.1	69.7
43	Genital discharge can be a symptom of an STI	True	**25.2**	25.2	6.9	67.8
44	Genital itching can be a symptom of an STI	True	**31.5**	31.5	6.0	62.4
45	Lower abdominal pain can be a symptom of an STI	True	**9.0**	9.0	20.8	70.2
46	Painless sores on the genitals can be a symptom of an STI	True	**19.9**	19.9	6.8	73.4
47	Infertility can be a consequence of an STI	True	**19.7**	19.7	11.0	69.3
48	Blindness can be a consequence of an STI	True	**31.5**	31.5	4.2	64.3
49	Headache can be a symptom of an STI	False	**10.8**	21.2	10.8	68.0
50	Haematuria (blood in the urine) can be a symptom of an STI	True	**27.4**	27.4	6.0	66.7
51	Impotence can be a consequence of an STI	False	**15.7**	11.3	15.7	73.0

Section G. STI prevention, HIV/AIDS and HPV vaccination

#	Questionnaire item	Correct answer	Correct (%)	Yes (%)	No (%)	Don't know (%)
52	Many people with an STI have no symptoms	True	**21.7**	21.7	7.1	71.2
53	Using oral contraceptives reduces the risk of acquiring an STI	False	**16.9**	18.0	16.9	65.1
54	Using condoms during intercourse reduces the risk of acquiring an STI	True	**48.3**	48.3	3.3	48.4
55	Having sex with multiple partners increases the risk of acquiring an STI	True	**46.2**	46.2	3.7	50.1
56	Untreated STIs can lead to death	True	**34.8**	34.8	5.4	59.8
57	Condoms protect against STIs 100% of the time	False	**45.0**	9.4	45.0	45.6
58	AIDS causes HIV	False	**12.6**	26.2	12.6	61.2
59	HIV causes AIDS	True	**30.6**	30.6	7.4	61.9
60	The HPV vaccine is recommended only for females	False	**14.1**	39.7	14.1	46.2

#	Questionnaire item	Correct answer	Correct (%)	Yes (%)	No (%)	Don't know (%)
61	After the first sexual intercourse it is too late to be vaccinated against HPV	False	**9.1**	41.0	9.1	49.9
62	The HPV vaccine is now also recommended for males	True	**52.5**	52.5	2.6	44.9
63	Vaccination is the most effective method to prevent HPV infection	True	**50.7**	50.7	5.8	43.5

Part 4. Attitudes, intentions and information sources

Attitude items were rated on a three-point scale (1 = disagree / no, 2 = uncertain, 3 = agree / yes). These items have no "correct" answer; the full distribution of responses is reported.

Q1. If you were HIV-positive, you would tell no one

Response	n	%
No	683	55.8
Don't know	332	27.1
Yes	209	17.1

Q2. If you were HIV-positive, you would tell your family doctor

Response	n	%
Yes	1102	90.0
Don't know	94	7.7
No	28	2.3

Q3. If you were HIV-positive, you would tell your best friend

Response	n	%
Yes	817	66.7
Don't know	241	19.7
No	166	13.6

Q4. If you were HIV-positive, you would tell your partner

Response	n	%
Yes	1062	86.8
Don't know	123	10.0
No	39	3.2

Q5. If you were HIV-positive, you would tell your family

Response	n	%
Yes	957	78.2
Don't know	188	15.4
No	79	6.5

Q6. If you were HIV-positive, you would have no problem telling everyone

Response	n	%
Don’t know	524	42.8
No	430	35.1
Yes	270	22.1

Q7. If you contracted an STI, would you inform your partner?

Response	n	%
Yes	698	57.0
Don’t know	495	40.4
No	31	2.5

Q8. Do you know the ideal age range to receive the HPV vaccine?

Response	n	%
Disagree / No	427	34.9
Don’t know	399	32.6
Any age	272	22.2
18–21 years	126	10.3

Q9. Are you aware that the vaccine is currently free for girls of a given age?

Response	n	%
No	693	56.6
Yes	531	43.4

Q10. Are you aware that the HPV vaccine is given in two or three doses?

Response	n	%
No	652	53.3
Yes	572	46.7

Q11. STIs are not something I worry about

Response	n	%
Agree / Yes	516	42.2
Disagree / No	440	35.9
Uncertain	268	21.9

Q12. I would like to receive the HPV vaccine myself

Response	n	%
Agree / Yes	749	61.2
Uncertain	392	32.0
Disagree / No	83	6.8

Q13. I have already received the HPV vaccine

Response	n	%
Uncertain	507	41.4

Response	n	%
Agree / Yes	463	37.8
Disagree / No	254	20.8

Q14. I have completed all doses of the HPV vaccine

Response	n	%
Uncertain	612	50.0
Agree / Yes	354	28.9
Disagree / No	258	21.1

Q15. The HPV vaccine allows us to stay safe and healthy

Response	n	%
Agree / Yes	734	60.0
Uncertain	411	33.6
Disagree / No	79	6.5

Q16. My parents decide whether I get the HPV vaccine

Response	n	%
Agree / Yes	427	34.9
Disagree / No	409	33.4
Uncertain	388	31.7

Q17. The cost of the vaccine discourages me

Response	n	%
Disagree / No	605	49.4
Uncertain	516	42.2
Agree / Yes	103	8.4

Q18. The protection the HPV vaccine would give me encourages me to get it

Response	n	%
Agree / Yes	728	59.5
Uncertain	419	34.2
Disagree / No	77	6.3

Q19. Do you consider your knowledge of STIs satisfactory?

Response	n	%
Uncertain	450	36.8
Disagree / No	415	33.9
Agree / Yes	359	29.3

Q20. Where did you learn about the existence of STIs?

Response	n	%
internet	168	13.7

Response	n	%
scuola	135	11.0
non ho informazioni	116	9.5
scuola;internet	47	3.8
scuola;amici;internet	32	2.6
amici;internet	31	2.5
amici	29	2.4
tv o radio;internet	26	2.1
nessuno;non ho informazioni	23	1.9
famiglia;amici;internet	19	1.6
None	19	1.6
famiglia	18	1.5
famiglia;internet	18	1.5
scuola;libri;opuscoli informativi;medico curante;riviste pubblicitarie;giornali;tv o radio;famiglia;amici;internet	17	1.4
tv o radio	16	1.3
scuola;libri;internet	14	1.1
scuola;famiglia	14	1.1
scuola;famiglia;amici;internet	14	1.1
scuola;tv o radio;internet	14	1.1
scuola;libri	12	1.0
tv o radio;amici;internet	12	1.0
scuola;tv o radio;amici;internet	12	1.0
Other / combined responses	418	34.2

Q21. Which STI do you think is the most common?

Response	n	%
HIV/AIDS	440	35.9
HIV/AIDS;HPV	148	12.1
herpes simplex	60	4.9
HPV	50	4.1
HIV/AIDS;herpes simplex	34	2.8
HIV/AIDS;herpes simplex;HPV	34	2.8
sifilide;HIV/AIDS;HPV	23	1.9
epatite b	23	1.9
sifilide	23	1.9
gonorrea	19	1.6
sifilide;HIV/AIDS	18	1.5
HIV/AIDS;clamidia	17	1.4
gonorrea;sifilide;HIV/AIDS;herpes simplex;clamidia;HPV;epatite c;epatite b	17	1.4
clamidia	15	1.2
HIV/AIDS;epatite c;epatite b	15	1.2

Response	n	%
HIV/AIDS;HPV;epatite c;epatite b	12	1.0
herpes simplex;HPV	12	1.0
Other / combined responses	264	21.6

Q22. I need more information about HPV infection

Response	n	%
Agree / Yes	909	74.3
Uncertain	247	20.2
Disagree / No	68	5.6

Q23. I need more information about the HPV vaccine

Response	n	%
Agree / Yes	864	70.6
Uncertain	264	21.6
Disagree / No	96	7.8

Note. *Questionnaire items were translated from the original Italian version. In Parts 1, 2 and 4, response categories with a frequency below 1% have been grouped as "Other / combined responses" for clarity, mainly multiple-selection answers. Percentages are computed on all 1,224 respondents.*
