# Supplementary material for: Knowledge, Attitudes, and Practices (KAP) Regarding Sexually Transmitted Infections, Human Papillomavirus, and HPV-Related Oropharyngeal Cancer Among Italian Adolescents: A Cross-Sectional Study
Source: Dent J (Basel). 2026 Aug 5;14(8):486. doi: 10.3390/dj14080486 (PMC13510924; doi:10.3390/dj14080486)
Supplement: Supplementary file 1 [file dentistry-14-00486-s001.zip › Supplementary_Material_S4.pdf]

Supplementary Material S4

Item-level comparison of correct-response rates by sex, with FDR-adjusted p-values

For each of the 63 knowledge items, the table reports the percentage of correct responses in girls and boys, the unadjusted chi-square p-value, and the p-value after Benjamini–Hochberg false-discovery-rate (FDR) correction across all 63 comparisons. These item-level comparisons are exploratory. Of the 36 differences significant at the nominal level, 25 remained significant after FDR correction, including awareness of HPV-related oropharyngeal cancer (item 6; FDR-adjusted p = 0.012). Rows significant after correction are shaded.

No.	Knowledge item	Girls %	Boys %	p (raw)	p (FDR)	Sig. (FDR)
1	HPV is a sexually transmitted virus	73.5	62.7	<0.001	**<0.001**	**Yes**
2	HPV infection is preventable	52.5	47.5	0.094	0.148	No
3	HPV infection occurs only in females	9.5	4.5	0.002	**0.006**	**Yes**
4	HPV can cause cancer of the female genital organs	32.0	20.8	<0.001	**<0.001**	**Yes**
5	HPV can cause cancer	32.2	25.0	0.008	**0.023**	**Yes**
6	HPV can also cause cancer of the mouth and throat (oropharynx)	17.3	11.2	0.003	**0.012**	**Yes**
7	HPV infection can cause cervical cancer	25.5	12.3	<0.001	**<0.001**	**Yes**
8	HPV can cause genital warts (condylomas)	9.5	6.4	0.071	0.115	No
9	Most HPV infections clear spontaneously	8.2	6.8	0.446	0.511	No
10	HPV can be treated with antibiotics	16.7	10.4	0.002	**0.008**	**Yes**
11	HIV/AIDS is transmitted through sexual intercourse	82.4	85.6	0.153	0.229	No
12	HIV/AIDS is transmitted through blood transfusion	54.6	53.0	0.631	0.685	No
13	HIV/AIDS is transmitted through needle sharing	60.0	58.5	0.652	0.697	No
14	HIV/AIDS is transmitted through food sharing	58.8	52.3	0.027	0.061	No
15	HIV/AIDS is transmitted through clothing sharing	2.6	5.1	0.033	0.068	No
16	HIV/AIDS is transmitted from mother to fetus	39.3	30.3	0.001	**0.006**	**Yes**
17	HIV/AIDS is transmitted through kissing (saliva)	30.3	29.5	0.832	0.832	No
18	Gonorrhoea is a sexually transmitted infection	22.6	23.7	0.698	0.733	No
19	Syphilis is a sexually transmitted infection	39.4	33.5	0.039	0.073	No
20	Genital herpes (herpes simplex) is a sexually transmitted infection	68.0	52.1	<0.001	**<0.001**	**Yes**
21	Tuberculosis is a sexually transmitted infection	11.1	15.5	0.027	0.061	No
22	Trichomoniasis is a sexually transmitted infection	9.2	7.2	0.258	0.346	No
23	Asthma is a sexually transmitted infection	3.2	5.9	0.034	0.068	No
24	HIV/AIDS is a sexually transmitted infection	79.0	81.2	0.377	0.449	No

25	Chlamydia is a sexually transmitted infection	33.2	21.8	<0.001	**<0.001**	**Yes**
26	Hepatitis B is a sexually transmitted infection	23.0	19.9	0.217	0.310	No
27	Hepatitis C is a sexually transmitted infection	17.3	19.9	0.285	0.362	No
28	STIs can be caused by bacteria	36.5	26.3	<0.001	**<0.001**	**Yes**
29	STIs can be caused by viruses	49.6	45.5	0.165	0.242	No
30	STIs can be caused by parasites	19.5	15.0	0.047	0.084	No
31	STIs can be caused by poor female hygiene	39.4	33.3	0.033	0.068	No
32	STIs can be caused by poor male hygiene	38.6	32.6	0.036	0.068	No
33	STIs can be caused by use of unclean water	24.6	18.9	0.022	0.054	No
34	STIs can be caused by having sex during menstruation	10.3	12.5	0.275	0.361	No
35	STIs are transmitted through sexual intercourse	50.5	47.9	0.402	0.469	No
36	STIs are transmitted through contact with body fluids	21.3	18.8	0.315	0.381	No
37	STIs are transmitted through oral sex	15.4	14.8	0.814	0.827	No
38	STIs are transmitted from mother to child	22.7	20.1	0.301	0.371	No
39	STIs are transmitted through coughing and colds	2.9	7.8	<0.001	**<0.001**	**Yes**
40	STIs are transmitted through ordinary social contact	3.2	4.5	0.287	0.362	No
41	STIs are transmitted through kissing	17.8	15.2	0.258	0.346	No
42	Genital ulcers can be a symptom of an STI	28.7	22.9	0.028	0.061	No
43	Genital discharge can be a symptom of an STI	30.0	19.1	<0.001	**<0.001**	**Yes**
44	Genital itching can be a symptom of an STI	38.9	22.0	<0.001	**<0.001**	**Yes**
45	Lower abdominal pain can be a symptom of an STI	10.8	6.6	0.016	**0.041**	**Yes**
46	Painless sores on the genitals can be a symptom of an STI	21.1	18.2	0.232	0.325	No
47	Infertility can be a consequence of an STI	21.5	17.0	0.059	0.101	No
48	Blindness can be a consequence of an STI	36.5	24.4	<0.001	**<0.001**	**Yes**
49	Headache can be a symptom of an STI	11.5	10.0	0.472	0.532	No
50	Haematuria (blood in the urine) can be a symptom of an STI	32.3	20.6	<0.001	**<0.001**	**Yes**
51	Impotence can be a consequence of an STI	16.2	14.8	0.562	0.621	No
52	Many people with an STI have no symptoms	24.3	18.0	0.010	**0.028**	**Yes**
53	Using oral contraceptives reduces the risk of acquiring an STI	19.5	13.3	0.005	**0.016**	**Yes**
54	Using condoms during intercourse reduces the risk of acquiring an STI	53.9	41.5	<0.001	**<0.001**	**Yes**

55	Having sex with multiple partners increases the risk of acquiring an STI	52.0	38.8	<0.001	**<0.001**	**Yes**
56	Untreated STIs can lead to death	37.0	32.4	0.109	0.168	No
57	Condoms protect against STIs 100% of the time	52.3	35.8	<0.001	**<0.001**	**Yes**
58	AIDS causes HIV	10.9	14.6	0.068	0.112	No
59	HIV causes AIDS	31.1	30.3	0.799	0.825	No
60	The HPV vaccine is recommended only for females	16.3	11.2	0.014	**0.037**	**Yes**
61	After the first sexual intercourse it is too late to be vaccinated against HPV	7.1	11.4	0.014	**0.037**	**Yes**
62	The HPV vaccine is now also recommended for males	56.0	48.1	0.007	**0.023**	**Yes**
63	Vaccination is the most effective method to prevent HPV infection	53.4	47.5	0.048	0.084	No
